# The antiviral state has shaped the CpG composition of the vertebrate interferome to avoid self-targeting

**DOI:** 10.1371/journal.pbio.3001352

**Published:** 2021-09-07

**Authors:** Andrew E. Shaw, Suzannah J. Rihn, Nardus Mollentze, Arthur Wickenhagen, Douglas G. Stewart, Richard J. Orton, Srikeerthana Kuchi, Siddharth Bakshi, Mila Rodriguez Collados, Matthew L. Turnbull, Joseph Busby, Quan Gu, Katherine Smollett, Connor G. G. Bamford, Elena Sugrue, Paul C. D. Johnson, Ana Filipe Da Silva, Alfredo Castello, Daniel G. Streicker, David L. Robertson, Massimo Palmarini, Sam J. Wilson

**Affiliations:** 1 MRC-University of Glasgow Centre for Virus Research (CVR), Glasgow, United Kingdom; 2 The Pirbright Institute, Woking, United Kingdom; 3 Institute of Biodiversity, Animal Health and Comparative Medicine, University of Glasgow, Glasgow, United Kingdom; Fred Hutchinson Cancer Research Center, UNITED STATES

## Abstract

Antiviral defenses can sense viral RNAs and mediate their destruction. This presents a challenge for host cells since they must destroy viral RNAs while sparing the host mRNAs that encode antiviral effectors. Here, we show that highly upregulated interferon-stimulated genes (ISGs), which encode antiviral proteins, have distinctive nucleotide compositions. We propose that self-targeting by antiviral effectors has selected for ISG transcripts that occupy a less self-targeted sequence space. Following interferon (IFN) stimulation, the CpG-targeting antiviral effector zinc-finger antiviral protein (ZAP) reduces the mRNA abundance of multiple host transcripts, providing a mechanistic explanation for the repression of many (but not all) interferon-repressed genes (IRGs). Notably, IRGs tend to be relatively CpG rich. In contrast, highly upregulated ISGs tend to be strongly CpG suppressed. Thus, ZAP is an example of an effector that has not only selected compositional biases in viral genomes but also appears to have notably shaped the composition of host transcripts in the vertebrate interferome.

## Abstract 2

Our cells are poised to combat viral infection through antiviral effectors. This study proposes that as well as targeting viral RNAs, antiviral effectors sometimes target host mRNAs too; over millions of years, this has selected for compositional biases in the host’s transcriptional response to virus infection.

## Introduction

Vertebrates have evolved a multitude of strategies to sense invading pathogens and deploy the appropriate immune defenses. A common outcome of pathogen sensing is the secretion of type I interferons (IFNs), which upregulate hundreds of interferon-stimulated genes (ISGs) [1–4]. Many ISGs interfere with viral replication, creating a hostile antiviral state within the IFN-stimulated cell [3].

The replication of every virus involves at least 1 viral RNA, and these molecules are frequent targets of both antiviral sensors and antiviral effectors [5]. To sense or target viral RNAs, host factors must possess the ability to discriminate self-RNAs from nonself-RNAs [5], and, to achieve this, the host exploits molecular features that are rare or absent in host transcripts. One of these features is the CpG dinucleotide (a cytosine base followed by a guanine), which is remarkably underrepresented in vertebrate genomes [6]. Synthetic viruses with enriched CpG content are severely attenuated in human cells as they succumb to CpG-targeting host defenses [7–10]. The zinc-finger antiviral protein (ZAP), an ISG, has evolved to contain a binding pocket that can accommodate a CpG dinucleotide, but no other dinucleotide [11]. ZAP binds to specific CpGs in viral RNAs and can target their degradation [10,12,13]. ZAP, and its binding partner TRIM25 [14,15], possess no intrinsic nuclease activity and mediate RNA degradation by recruiting cofactors such as the putative nuclease KHNYN [16]. Crucially, not all CpGs are equally targeted by ZAP, and the context of the CpGs, as opposed to the number of CpGs, appears to define whether a particular CpG or transcript is targeted by ZAP [10,17,18]. Nonetheless, the number of CpGs is a useful feature, as increased CpG frequency increases the likelihood that a CpG is presented in a context recognised by ZAP. Although the contextual features underlying ZAP targeting are not fully understood, it has recently been proposed that the CpG motif must be presented in a region of single-stranded RNA [19].

It is widely believed that the majority of RNA viruses infecting vertebrates have evolved to possess a relatively low CpG content [20] to escape CpG-targeting defenses [8–10,21], and DNA viruses might also suppress their transcribed CpG content to escape ZAP [22]. However, the extent to which antiviral defenses have influenced the composition of the host genome is unknown. Both ZAP and TRIM25 are evolutionarily conserved “core” ISGs [4]. We therefore hypothesised that ISGs, which are highly upregulated during the IFN response, have been selected to contain fewer CpGs, as they have functioned in the presence of abundant ZAP for over 300 million years [4]. Similarly, although most studies of the transcriptional response to IFN (the “interferome”) focus on ISGs, the abundance of many host transcripts actually decreases following exposure to IFNs [1,2,4], and these genes are classified as interferon-repressed genes (IRGs). We thus additionally hypothesised that cellular mRNAs with abundant CpGs are targeted by ZAP during the IFN response. Thus, ZAP targeting of self-RNAs could be the mechanism through which some IRGs are downregulated.

Here, we show that across multiple species, ISG mRNAs are compositionally distinct, with the most important difference being that the CpG content of ISG transcripts is typically strongly suppressed, whereas IRGs tend to have a relatively high CpG content. We further reveal that IFN-stimulated ZAP expression appears to mediate the IFN-induced repression of multiple host transcripts. We propose that the antiviral state targets specific CpGs in mRNAs, and over millions of years, this has driven highly upregulated ISGs to possess very low CpG contents. Together, our data indicate that the impact of ZAP on compositional bias extends far beyond viruses to include significant impacts on the composition of the vertebrate interferome.

## Results

### The host transcriptional response to interferons has a CpG bias

**Fig 1:**
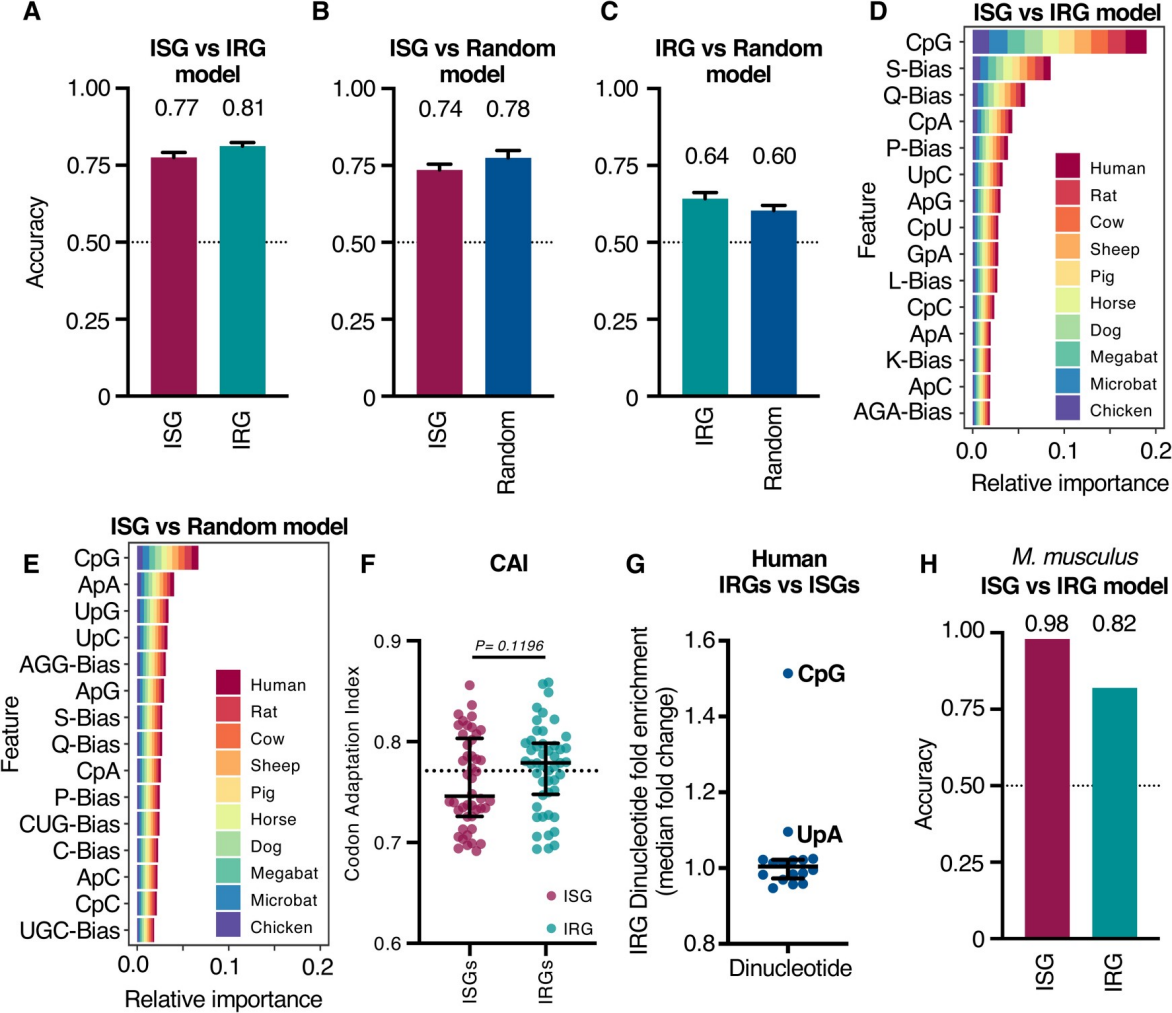
Compositional features predict expression class following IFN treatment. **(A–C)** Accuracy of classifiers trained to distinguish the top 50 most DE ISGs from the top 50 most DE IRGs (A), ISGs from 50 random genes (from each species) (B), or IRGs from 50 random genes (from each species) (C). Bars show the average proportion of genes in each class that were accurately identified across 50 replicates of 5-fold cross-validation, while error bars show the region containing 95% of observed accuracy values. Dashed lines indicate the expected performance of a null (i.e., uninformative) model. **(D, E)** The 15 most important features used by classifiers to distinguish ISGs from IRGs (D) or ISGs from random genes (E). The equivalent panel with the features used by classifiers to distinguish IRGs from random genes is shown in S3 Fig. Feature importance was quantified for individual genes using the SHAP approach [23,24], before summing their magnitude across all genes from a given species. All classifiers were trained and evaluated on 500 genes from each class, representing the top 50 ISGs or IRGs, or 50 random genes, from each species. Three-letter codes of the form “CpG” indicate measures of dinucleotide composition, single letters followed by the word “bias” (e.g., S-Bias) indicate amino acid composition biases, and 3 letters followed by the word “bias” (e.g., AGA-Bias) indicate codon usage biases. **(F)** The CAI of the 50 most DE ISGs and IRGs calculated as described previously [25]. The CAI is a measure of optimal codon usage; a higher CAI indicates a more optimal usage of codons. The horizontal dotted line represents the median for all transcripts in the human genome. Statistical significance was assessed using the Wilcoxon rank sum test with continuity correction. **(G)** The fold change in median dinucleotide composition between the top 50 most DE ISGs and IRGs in humans (summarising S2 Fig). The underlying RNA-seq data analysed in (A–G) were our previously published open-access data [4]. Briefly, primary fibroblasts derived from human, rat, cow, sheep, pig, horse, dog, little brown bat, and chicken, as well as immortalised large flying fox cells, were treated with type I IFNs (1,000 U/ml universal IFN, 200 ng/ml canine IFNα, 1,000 U/ml porcine IFNα, or 200 ng/ml chicken IFNα) for 4 hours before being analysed using RNA-seq [4]. **(H)** Accuracy of classifiers trained to distinguish the top 50 most DE ISGs from the top 50 most DE IRGs in a microarray dataset from a species not used to develop the model (murine NIH 3T3 cells +/− 100 units IFN [26], extracted from the interferome database [2]). The underlying data from this figure are openly available (http://dx.doi.org/10.5525/gla.researchdata.1159). CAI, Codon Adaptation Index; DE, differentially expressed; IFN, interferon; IRG, interferon-repressed gene; ISG, interferon-stimulated gene; RNA-seq, RNA sequencing; SHAP, SHapley Additive exPlanations.

We initially examined the CpG content of human ISGs and IRGs in relation to their induction and repression in response to IFN treatment. Using open-access RNA sequencing (RNA-seq) data we previously generated using IFN-treated primary human fibroblasts [4], we observed that the most highly upregulated ISGs tended to possess a highly suppressed CpG content (S1 Fig). We therefore selected the 50 most upregulated and 50 most downregulated genes for subsequent analysis as this compositional bias appeared most extreme within these groups (S1 Fig). Because CpG abundance is just one feature, we also examined the compositional differences among the transcripts produced from these genes more generally (across 10 different species, alongside 50 randomly sampled, robustly expressed, but not differentially expressed [DE] genes from each species; S1 Table). For our gene lists, we again used a publicly available RNA-seq dataset that we previously generated using cells from these 10 species, which were stimulated with type I IFN under standardised conditions [4]. We trained 3 supervised machine learning classifiers to distinguish between (1) ISGs and IRGs; (2) ISGs and random genes; and (3) IRGs and random genes, based on 185 compositional features (encompassing dinucleotide, codon, and amino acid biases). Remarkably, across 50 replicates of 5-fold cross-validation, highly upregulated ISGs were reliably distinguished from the most repressed IRGs (>77% accuracy), and from random genes (with >74% accuracy), based upon their composition alone (Fig 1A and 1B; *N* = 1,000 in all cases, i.e., 50 genes from each of the 2 classes being distinguished, across 10 species). This is considerably better than the 50% accuracy expected by chance. Thus, even in the absence of important contextual features (such as the promoter region), responsivity to IFN was predictable based on the composition of the coding sequences (CDSs) alone. The majority of this signal seemed to originate from the ISGs, as IRGs were less reliably distinguished from random genes (accuracy >60%) (Fig 1C).

Strikingly, when the relative importance of each compositional feature was examined, the CpG content was the most definitive feature that distinguished ISGs from IRGs or from random genes across all 10 species used for the training (Fig 1D and 1E). Multiple other compositional differences were also utilised by the classifiers, suggesting that the selective pressures that sculpt the broader composition of ISGs may be distinct. Importantly, the distinctive composition of ISGs was not merely a consequence of the most upregulated ISGs using codons that are conducive to efficient protein expression. When the codon adaptation indices [25] of the most DE human ISGs and IRGs were compared, the ISGs tended to use similar or less optimal codons than the IRGs (Fig 1F). Thus, the compositional features of ISGs that enable efficient expression in the antiviral state appear distinct from those that simply promote efficient expression in unstimulated cells.

Because the CpG composition appeared to be the most defining feature of ISG transcripts, we analysed the dinucleotide composition of the most DE human ISGs and IRGs (S2 Fig). In accordance with the relative importance estimates (Fig 1D and 1E), CpG was the most variable dinucleotide, with IRGs containing approximately 50% more CpGs than ISGs (Fig 1G). As observed above, most of this signal appeared to be associated with ISGs. While CpG was among the most informative features in the classifier trained to distinguish IRGs from random genes, it was not the most important feature (S3 Fig). To examine the utility of the machine learning approach (Fig 1A–1E), using an interferome from a species not used to develop the model, we examined the ability of the model to correctly classify the 50 most DE mouse ISGs and IRGs [2,26]. Remarkably, the composition-based discriminatory power extended to a species not present during training, with 49 out of 50 mouse ISGs and 41 out of 50 mouse IRGs accurately classified using composition alone [2,26] (Fig 1H).

The relative importance of the CpG dinucleotide across all classifiers (Fig 1D and 1E, S3 Fig) led us to examine the CpG composition of the most DE ISGs and IRGs more closely. There are multiple methods for calculating CpG composition, and we have used a simple CpG frequency measurement (normalised to the length of the transcript) throughout. In addition, most experiments are also presented using CpG frequency normalised to the guanine–cytosine (GC) content of the transcript (equivalent to observed/expected), which is a standard method for calculating CpG composition.

**Fig 2:**
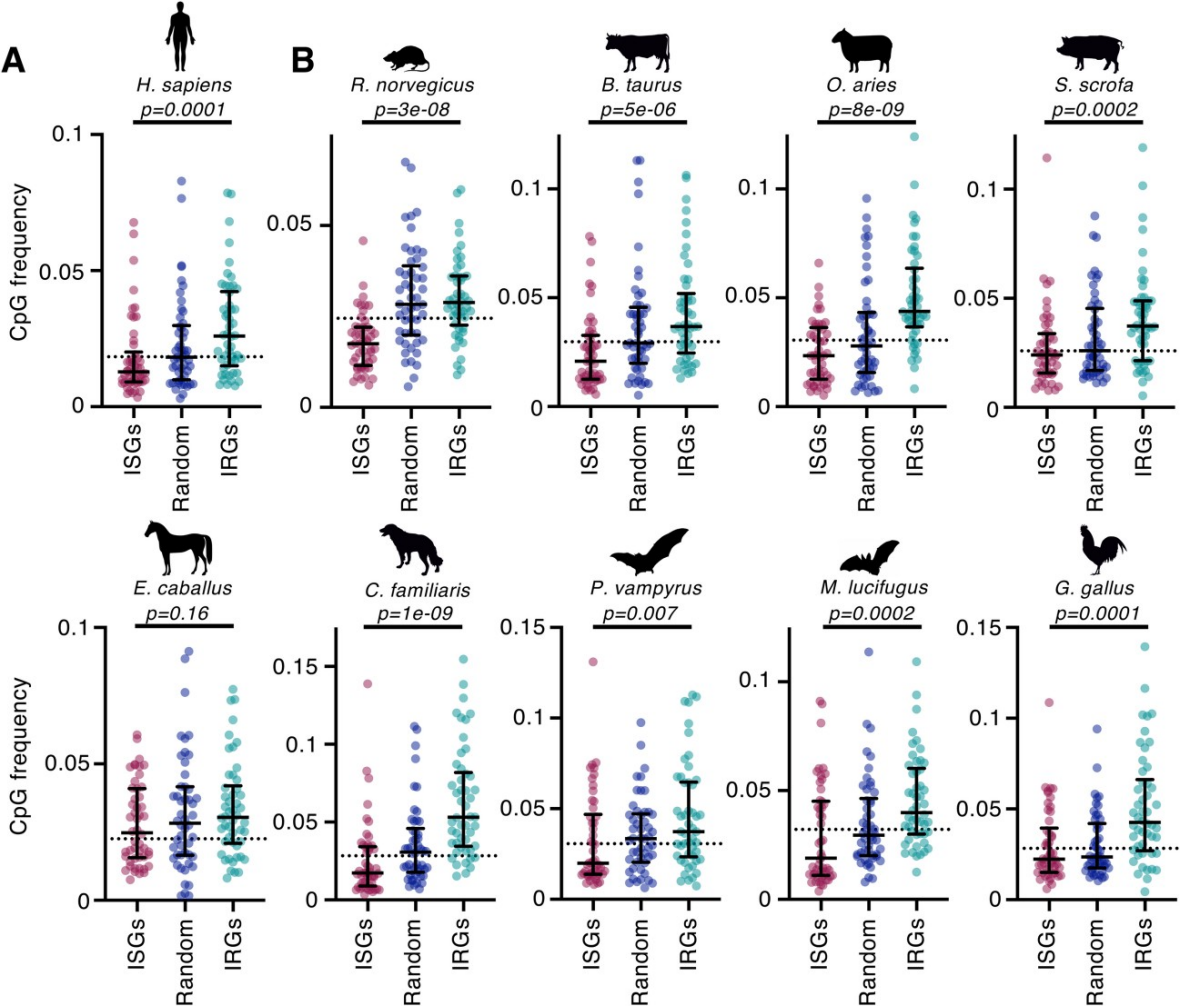
The vertebrate interferome has a CpG bias. **(A)** The length-normalised (cDNA) CpG frequency (see Materials and methods) of the top 50 most DE human ISGs and IRGs (ranked by mean Log_2_FC) is shown. The dashed line represents the median CpG frequency of all transcripts in the relevant genome, a random sample of non-DE genes is included for reference, and whiskers represent the median and interquartile range for the analysed group. **(B)** The interferomes of the remaining 9 vertebrate species are plotted as in (A). The underlying RNA-seq data used were previously published open-access data [4] and were also described in the Fig 1 legend. Significance was determined using the Wilcoxon rank sum test with continuity correction. The underlying data from this figure are openly available (http://dx.doi.org/10.5525/gla.researchdata.1159). DE, differentially expressed; IRG, interferon-repressed gene; ISG, interferon-stimulated gene; RNA-seq, RNA sequencing.

When we examined the CpG composition of the human interferome, we observed that the top 50 DE ISGs possessed significantly fewer CpGs than the corresponding group of 50 IRGs (Fig 2A). Importantly, ISGs tended to have even fewer CpGs than the median of all human transcripts, whereas IRGs tended to be relatively enriched in CpGs (Fig 2A). The observed difference in the CpG content of ISGs and IRGs remained strongly evident regardless of whether we considered the frequency of CpGs (Fig 2A) or the frequency of CpGs normalised to overall GC content (S4 Fig).

We next examined the CpG content of the most DE ISGs and IRGs from 9 additional species (encompassing over 300 million years of evolution). Interestingly, this evolutionary divergence has resulted in considerable variation in the overall levels of CpG suppression in each species. The most extreme example was the large flying fox (*Pteropus vampyrus*), whose median CpG frequency was approximately 65% higher than in the human genome (Fig 2A and 2B). Despite this variation, in every species, the median CpG content of the most DE IRGs was noticeably higher than the median of all transcripts in that species (Fig 2B). Moreover, the CpG content of IRGs was also significantly higher than that of ISGs in 8 of the 9 additional species and was highly significant in multiple species, such as dogs and sheep (Fig 2B). Notably, the median CpG content of the most DE canine IRGs was 3-fold higher than that of the corresponding ISGs. The exception to the overall trend was the horse, in which the CpG composition of IRGs remained marginally higher than that of ISGs (*p* = 0.16; Fig 2B). This weaker effect may represent a biological difference in the equine interferome, but it might also reflect the methods used to annotate the horse genome (as misannotated sequences could result in substantial errors in the calculated CpG composition). Notably, the overall trends were very similar when the normalised measure of CpG composition was used (S4 Fig). Overall, the observed dinucleotide bias in interferomes from divergent species suggests that this bias is an ancient property of vertebrate interferomes.

### Type I interferons exhibit extreme CpG suppression

**Fig 3:**
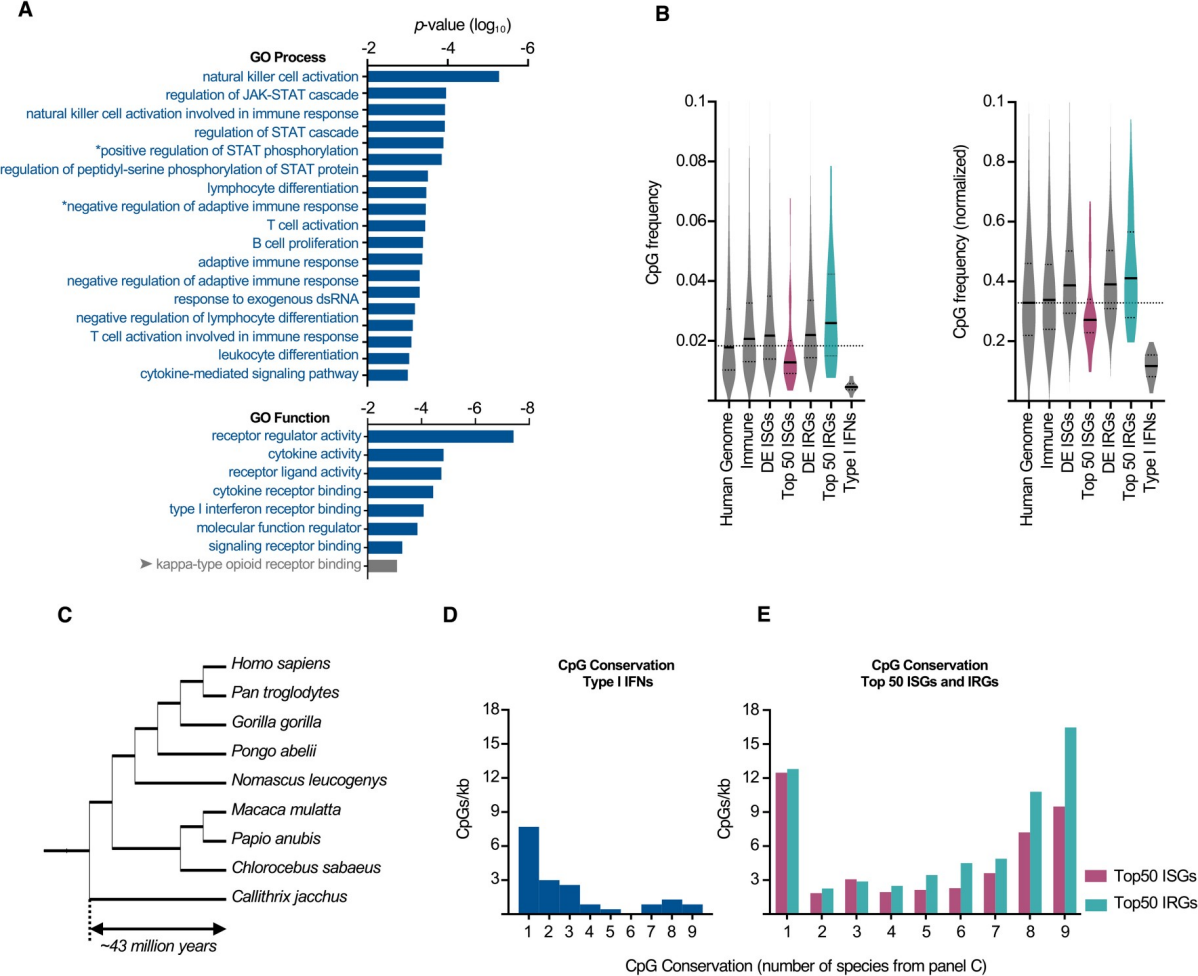
Type I IFNs exhibit extreme CpG suppression. **(A)** Significantly enriched GO processes and functions identified through GOrilla enrichment analysis of the 1,000 most CpG-suppressed cDNAs >100 bp (compared to all cDNAs >100 bp). **(B)** The CpG frequency in immune genes, ISGs, IRGs, and type I IFNs (for ISGs and IRGs, all significantly DE genes and the top 50 most DE genes are plotted). Matrices highlighting the significance (Kruskal–Wallis rank sum test) of potential comparisons in Fig 3B are displayed in S6 Fig. **(C)** A phylogenetic tree of the 9 primate species used to quantify CpG conservation in Figs 3D, 3E, and 5D. **(D, E)** Conservation is plotted as the number of CpGs per kb, binned by the number of species that possess that specific CpG, where 1:1 orthologs exist. Bin “1” represents CpGs present in only 1 of the species, whereas bin “9” represents CpGs conserved in all of the 9 species considered. CpG conservation is plotted for (D) type I IFNs (4 1:1 orthologs) and (E) top 50 ISGs (30 1:1 orthologs) and IRGs (34 1:1 orthologs). * Full GO process names: “positive regulation of peptidyl-serine phosphorylation of STAT protein” and “negative regulation of adaptive immune response based on somatic recombination of immune receptors built from immunoglobulin superfamily domains.” Arrow indicates a function enriched by genes other than the IFN genes IFNA2 and IFNA14. The underlying data from this figure are openly available (http://dx.doi.org/10.5525/gla.researchdata.1159). DE, differentially expressed; dsRNA, double-stranded RNA; GO, gene ontology; IFN, interferon; IRG, interferon-repressed gene; ISG, interferon-stimulated gene.

The observation that potently up-regulated ISGs tended to be strongly CpG suppressed led us to consider whether other genes playing pivotal roles in immunity might also share this property. We first calculated the dinucleotide frequency of every transcript in the human genome and confirmed the overall tendency for CpGs to be suppressed (S5 Fig). Then, in order to investigate the most CpG-suppressed genes, and determine if such genes shared any biological characteristics, we conducted gene ontology (GO) enrichment analysis [27] of the 1,000 human transcripts with the lowest CpG content (that were longer than 100 nucleotides in length). Remarkably, of all the overrepresented GO processes and functions, all but one function were involved in immune responses (Fig 3A). Importantly, nearly all the overrepresented terms were due to multiple type I IFN genes being among the 1,000 most CpG-suppressed genes, as observed previously [28]. GOrilla enrichment analysis of the same subset, in the absence of IFNα2 and IFNα14 (whose GO annotation and presence in the 1,000 most CpG suppressed likely accounted for these overrepresented terms), resulted in no significant overrepresentation of any immune processes (unpublished observations). Similar results were obtained when the normalised CpG frequency was considered (S5 Fig).

To explore whether extreme CpG suppression was a general property of immune genes, we considered the CpG content of 1,678 known immune genes (collated by ImmPort [29]), together with all significantly DE ISGs and IRGs we described previously [4]. The CpG content of the “immune genes,” ISGs and IRGs, were very similar to when all transcripts in the genome were considered together (Fig 3B). Importantly, many genes are classified as significantly DE ISGs or IRGs, even though their transcript abundance varies only subtly following IFN stimulation (S1 Fig). These subtly modulated genes may not play a substantial role in IFN responses. In contrast, the CpG content of the 50 most DE ISGs and IRGs (who likely play a more important role in IFN responses) was noticeably different from the median of all human transcripts (Fig 3B). Moreover, in both cases, the CpG content was significantly different from that of all human transcripts (Fig 3B, S6 Fig). Importantly, the dinucleotide bias observed in the 50 most DE ISGs and IRGs is not observed in the whole interferome and is not present in most immune genes. Thus, the compositional bias has likely been selected for in genes whose expression is highly responsive to IFN stimulation.

In accordance with the GO analysis, the CpG content of type I IFN transcripts was remarkably low, and these genes were among the most CpG-suppressed transcripts in the entire human genome (Fig 3B). We therefore examined the CpG suppression in type I IFNs from a selection of the 10 species from Figs 1 and 2, whose type I IFN loci were well-defined [30]. Strong CpG suppression was also observed in mammalian type I IFN genes from cows, pigs, and large flying foxes (S7 Fig), despite the differential expansion of diverse IFN subtypes in these species. Again, a notable exception was the horse, where, similar to highly expressed ISGs, CpG suppression was not unusually strong (S7 Fig). In contrast, chicken type I IFNs were relatively CpG rich. These differences do not appear to be due to the different IFN subtypes, as IFNα is the predominant subtype in both humans and chickens, and IFNω is the most common subtype in cows and horses. Thus, CpG suppression in IFNs appears to be more evolutionarily heterogeneous than in highly expressed ISGs, where relatively high CpG suppression is uniformly observed.

We next considered the level of CpG conservation in the different gene classes where one-to-one orthologs existed in 9 primate species [31]. In addition to possessing very few CpGs, the CpGs in primate type I IFNs were also short-lived, with fewer than 1 CpG/kb present in the common ancestor being conserved in all 9 extant species (approximately 43 million years of divergence [32]) (Fig 3C and 3D). Similarly, CpGs appeared to be rapidly purged from ISGs, as IRGs contained approximately 70% more 43 million–year-old CpGs/kb than ISGs (Fig 3E). Thus, the most upregulated ISGs not only possess fewer CpGs (Fig 2), but these CpGs are also more rapidly lost (Fig 3E), consistent with stronger selection against CpGs in ISGs.

### The ZAP network targets specific host mRNAs and mediates interferon-induced repression of gene expression

**Fig 4:**
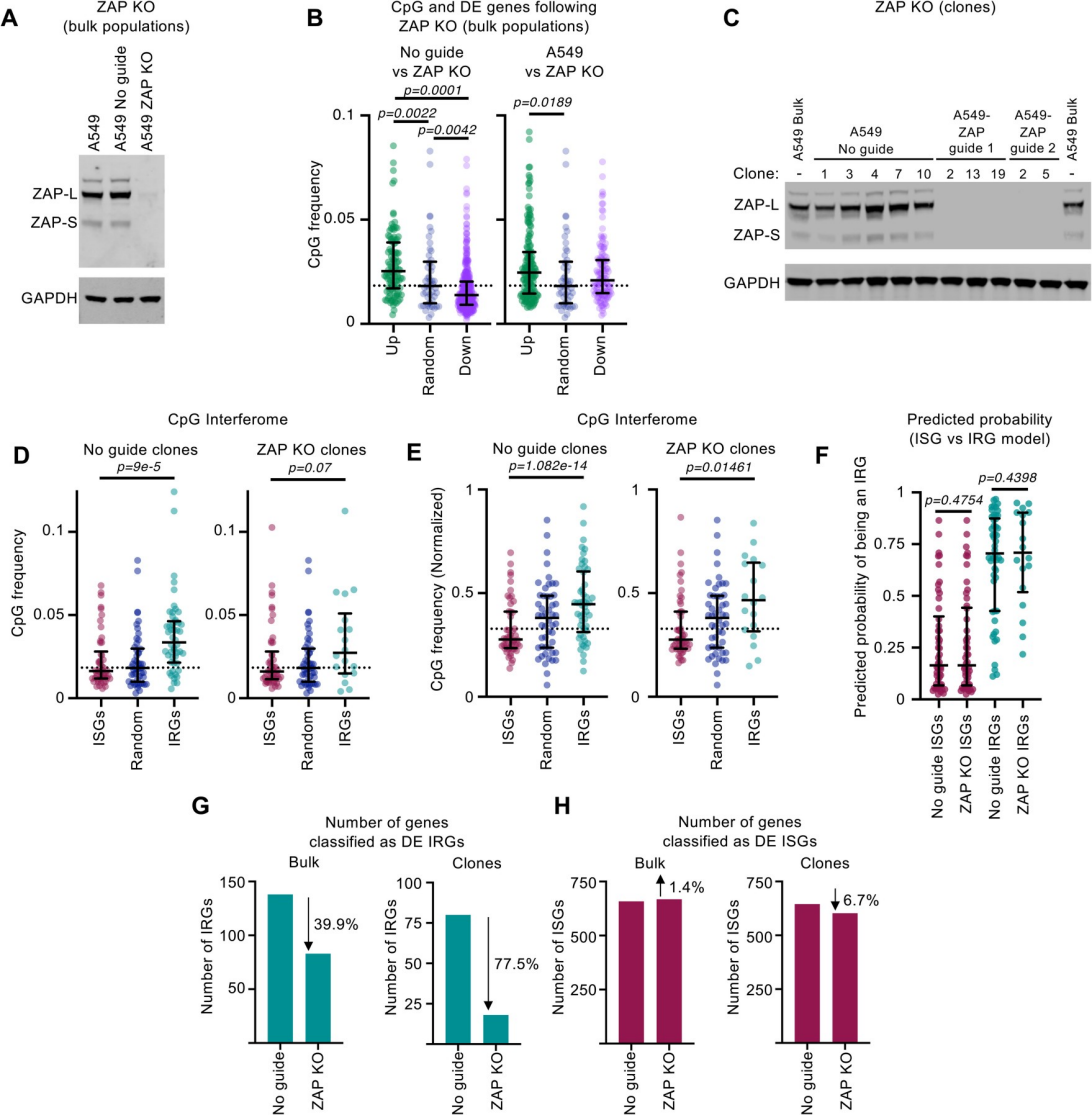
ZAP mediates the IFN-induced repression of a subset of IRGs. **(A)** WB analysis of ZAP and GAPDH expression in human “bulk populations” of A549 cells transduced with ZAP-targeting CRISPR sgRNAs or transduced Cas-9 expressing “No guide” controls. ZAP-L (PARP13.1) and ZAP-S (PARP13.2) bands are indicated [34]. **(B)** The CpG content of significantly DE genes (identified using RNA-seq in the absence of IFN using edgeR and an FDR <0.05), comparing triplicate “bulk” KO cells and transduced controls or unmodified cells. A random selection of 50 genes is included for comparison. Bars represent the median values, and whiskers represent the interquartile ranges. Horizontal dotted lines represent the median of all transcripts in the human genome. Significance was determined using a Kruskal–Wallis rank sum test (only significant comparisons are shown). **(C)** WB analysis (as in A) of clonal lines modified with ZAP-targeting CRISPR sgRNAs or parallel clonal lines derived from Cas-9 expressing transduced “No guide” controls. **(D, E)** The CpG content (D) or the normalised CpG content (E) of the 50 most significantly DE ISGs and IRGs (ranked by mean Log2FC), determined using RNA-seq of the 10 clones in (C) stimulated with 1,000 units/ml of IFNβ (4 hours) are shown, alongside 50 random genes. Where fewer than 50 significantly DE genes were detected, all significantly DE genes are plotted. Significance was assessed using the Wilcoxon rank sum test with continuity correction. **(F)** The predicted probability that the IRGs classified in the presence of ZAP or the absence of ZAP, from (D and E), are IRGs based on their nucleotide composition. Significance was assessed using the Kruskal–Wallis rank sum test (only insignificant comparisons are shown). **(G, H)** Numbers of significantly DE IRGs (G) or ISGs (H) from the RNA-seq of cells in either (A) (“bulk”) or (C) (“clones”) stimulated with 1,000 units/ml of IFNβ (4 hours). The underlying data from this figure are openly available (http://dx.doi.org/10.5525/gla.researchdata.1159). DE, differentially expressed; FDR, false discovery rate; IFN, interferon; IRG, interferon-repressed gene; ISG, interferon-stimulated gene; KO, knockout; RNA-seq, RNA sequencing; sgRNA, single-guide RNA; WB, western blot; ZAP, zinc-finger antiviral protein.

The reduced abundance and rapid evolutionary turnover of CpGs in strongly induced ISGs led us to consider whether ZAP (a CpG-targeting effector) might influence the transcript abundance of IFN-regulated genes. To investigate this possibility, we depleted ZAP from human cells using CRISPR/Cas-9. We opted to use A549 cells for these analyses as previous work has shown that the replication of an influenza A virus engineered to possess an elevated CpG content is severely restricted in these cells [9]. We observed that ZAP protein expression was efficiently depleted when “bulk” A549 populations, transduced with vectors encoding ZAP-targeting guide RNAs, were compared to Cas-9 expressing transduced controls without a ZAP guide (Fig 4A). To examine the effect ZAP depletion had on the A549 transcriptome, we carried out RNA-seq analysis of these “bulk” populations. We opted to limit our analysis to bulk populations in this instance as clonal variants (from knockout [KO] clones) often have distinct transcriptomic signatures that could obscure the signal from ZAP depletion. Comparison of the transcriptomes of ZAP KO and “No guide” controls revealed a number of significantly DE genes. Notably, the CpG content of transcripts whose expression increased following ZAP depletion tended to be higher than the median CpG content of all transcripts in the genome (Fig 4B). This observation is consistent with the notion that ZAP-mediated RNA surveillance might increase the turnover of CpG-rich mRNAs in resting cells. Interestingly, a large number of genes were downregulated following ZAP depletion (as has been reported previously [33]). We speculate that multiple genes involved in transcriptional regulation (possibly upregulated following ZAP depletion) might indirectly downmodulate these transcripts.

To investigate whether ZAP targeting of host mRNAs might be involved in the dinucleotide bias observed in ISGs and IRGs, we generated 5 clonal A549 complete ZAP KO cell lines (Fig 4C) and determined their transcriptional response to IFN alongside 5 clones of transduced “No guide” clonal control cell lines generated in parallel (Fig 4C). In addition, we also defined the interferome of our bulk KO cells. We then analysed the CpG content of the significantly DE genes. ISGs tended to possess fewer CpGs than IRGs, regardless of the presence or absence of ZAP (Fig 4D and 4E). Similarly, the composition of IRGs in IFN-stimulated cells with and without ZAP was remarkably similar in terms of overall CpG content (Fig 4D and 4E). Moreover, the ISG versus IRG classifier did not appear to identify ZAP-independent IRGs any less reliably than the top 50 IRGs (Fig 4F). Thus, our classifier does not specifically detect the CpGs targeted by ZAP. Strikingly, however, fewer genes were repressed following IFN treatment when ZAP was depleted. The total number of genes classified as IRGs was approximately 40% lower in bulk ZAP KO cells and was approximately 75% lower in ZAP KO clones, suggesting that ZAP is involved in reducing the abundance of a sizable subset of IRG transcripts (Fig 4G). Conversely, ZAP KO cells had a similar number of ISGs to control cells (Fig 4H). Importantly, the continued presence of IRGs in the absence of ZAP expression reveals that not all IRGs are dependent upon ZAP.

**Fig 5:**
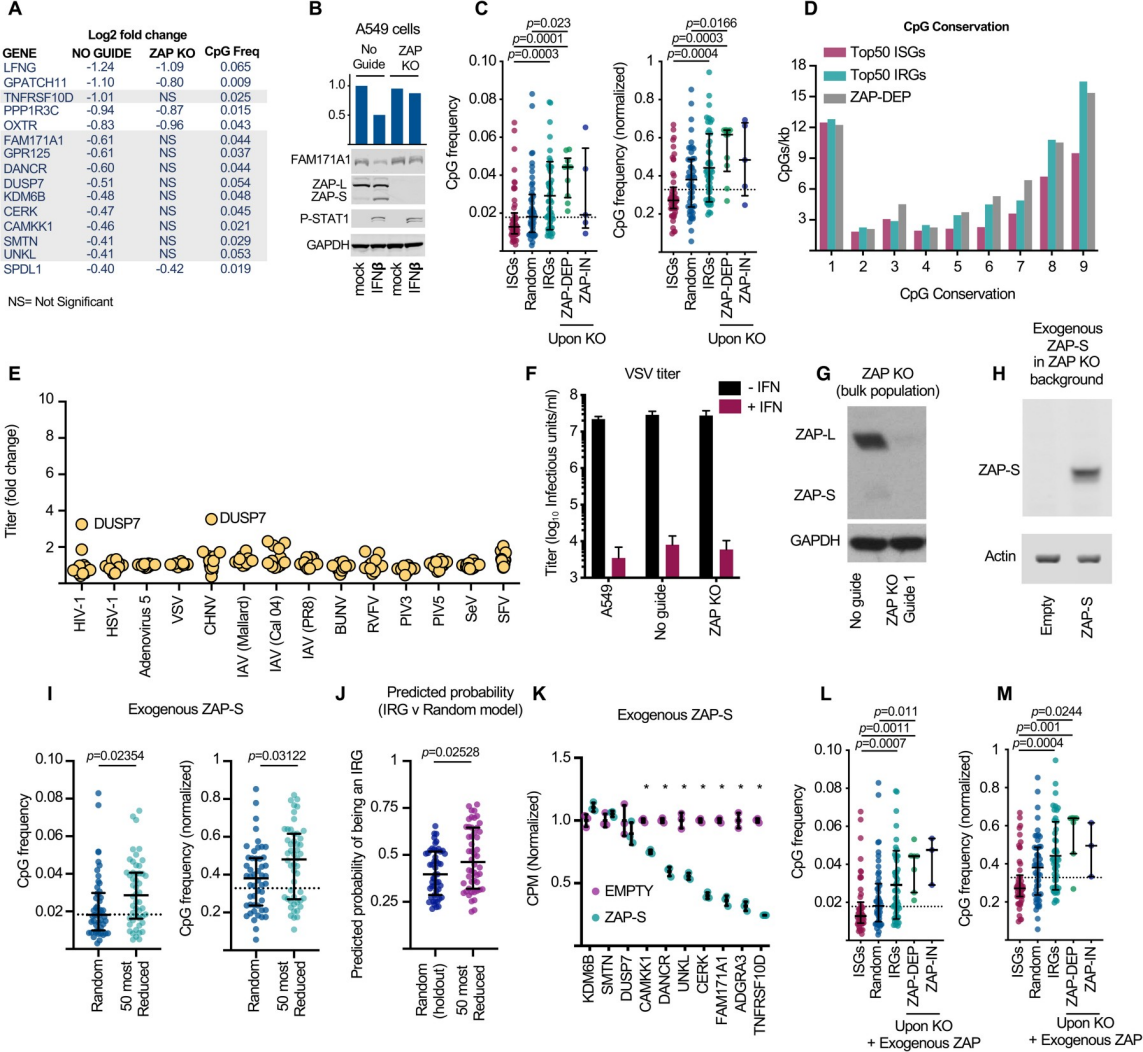
Identification of host transcripts consistently targeted by IFN-stimulated ZAP. **(A)** The identity and differential expression of the 15 IRGs identified in Shaw et al. [4], as well as in bulk and clone NO GUIDE controls (derived from the overlapping populations, left-hand side of panel B in S8 Fig). Shaded genes represent IRGs whose repression is not observed following ZAP KO. **(B)** WB of FAM171A1, ZAP, P-STAT1, and GAPDH in A549 NO GUIDE or ZAP KO clones stimulated with 1,000 units/ml of IFNβ (24 hours). **(C)** The CpG contents of 10 ZAP-dependent (ZAP-DEP) IRGs and 5 ZAP-independent (ZAP-IN) IRGs (identified in A) are shown alongside the most DE ISGs and IRGs from Fig 2A. Significance was assessed using the Kruskal–Wallis rank sum test; significant differences are shown (all comparisons listed in S9 Fig). **(D)** CpG conservation among 9 primate species (as in Fig 3C–3E) is plotted as the number of CpGs per kb, binned by the number of species that possess that specific CpG, where 1:1 orthologs exist (ZAP-DEP *n* = 5). “1” represents CpGs present in only 1 of the species, whereas “9” represents CpGs conserved in all of the 9 species considered (top 50 ISGs includes 30 1:1 orthologs and top 50 IRGs includes 34 1:1 orthologs). **(E)** The 10 putative ZAP targets (identified in A) encoded by lentiviral vectors were used to transduce human cells prior to serially diluted challenge with a GFP-expressing virus. The resulting titres were calculated using flow cytometry and normalised to the empty vector control. These data were plotted as the fold change in titre (y-axis) relative to the empty vector control cells (yellow circles represent each cDNA). **(F)** The impact of ZAP KO (see Materials and methods) on the replication of *Indiana vesiculovirus* in the presence and absence of IFNβ. **(G)** WB for ZAP in bulk A549 cells KO’d for ZAP (ZAP KO guide 1) compared to mock treated cells (No guide). **(H)** WB of A549 ZAP KO cells (guide 1, clone 2; Fig 4C) transduced with an empty lentiviral vector or a lentiviral vector encoding CRISPR-resistant ZAP-S. **(I)** The transcriptomes of the transduced cells in (H) were defined using RNA-seq and the CpG compositions of the 50 most downregulated transcripts are shown. Significance was determined using the Wilcoxon rank sum test with continuity correction. **(J)** The predicted probability that the most reduced transcripts in (I) are IRGs. A sample of 50 random genes (not used for training) are included as a comparator. Significance was determined as in (I). **(K)** The transcript abundance of the 10 ZAP targets identified in (A) in the cells from (H) and (I) is shown. **(L)** The CpG frequency and **(M)** normalised CpG frequency of the 7ZAP-DEP IRGs and 5 ZAP-independent (ZAP-IN) IRGs (identified in A and J) are shown alongside the most DE ISGs and IRGs from Fig 2A. Significance was assessed as in (C). The dashed line in (C), (I), (L) and (M) represents the median CpG frequency of all transcripts in the genome, a random sample of non-DE genes is included for reference, and whiskers represent the median and interquartile range for the analysed group. The underlying data from this figure are openly available (http://dx.doi.org/10.5525/gla.researchdata.1159). BUNV, *Bunyamwera orthobunyavirus*; CHNV, *Chandipura vesiculovirus*; DE, differentially expressed; HIV-1, *Human immunodeficiency virus 1*; HSV-1, *Human alphaherpesvirus 1* (formerly known as herpes simplex virus 1); IAV, Influenza A virus; IFN, interferon; IRG, interferon-repressed gene; ISG, interferon-stimulated gene; KO, knockout; P-STAT1, phosphorylated STAT1; PIV-3, *Human respirovirus 3* (formerly parainfluenza virus 3); PIV-5, *Mammalian orthorubulavirus 5* (formerly parainfluenza virus 5 or simian virus 5); RNA-seq, RNA sequencing; RVFV, *Rift Valley fever phlebovirus*; SeV, *Murine respirovirus* (formerly Sendai virus); SFV, *Semliki Forest virus*; VSV, *Indiana vesiculovirus* (formerly vesicular stomatitis virus); WB, western blot; ZAP, zinc-finger antiviral protein; ZAP-DEP, ZAP-dependent.

Interestingly, the identity of the IRGs in bulk and ZAP KO clones was far more variable than that of ISGs in the equivalent cells (S8 Fig). This likely reflects the fact that ISGs are very strongly upregulated (sometimes >1,000-fold), whereas IRGs are seldom repressed more than 2-fold, both here (S1 Fig) and in previous studies [1,2,4]. Moreover, downmodulation of a transcript is dependent on sufficient expression of that transcript prior to IFN stimulation, making the IRG class more dependent on cell state, lineage, and culture conditions than ISGs. Thus, to identify putative ZAP targets, we focused on the IRGs that were most consistently downregulated across our datasets. First, we filtered our A549 transcriptomic data to include only DE genes also observed in primary human cells [4] (S8 Fig). Among these genes, we selected those that were also IRGs, in both A549 “bulk” and clonal control cells (present in the overlapping populations on the left-hand side of panel B, S8 Fig). This approach identified 15 genes that were significantly downregulated in all 3 RNA-seq experiments (Fig 5A). Remarkably, when ZAP was knocked out, two-thirds of these consistent IRGs were no longer significantly downregulated by IFN in any ZAP KO condition (either “bulks” or individual “clones”) (Fig 5A). The functions of these IRGs are summarised in S2 Table. This observed change in transcript abundance might also be reflected at the protein level as one readily detectable antigen appeared similarly repressed by IFN in a ZAP-dependent fashion (Fig 5B). These observations suggest that ZAP plays a role in the downregulation of a subset of IRGs. Notably, the only previously well-defined cellular target of ZAP, TNFRSF10D [33], is a known IRG [2,4] (S9 Fig) and was among the genes that we identified using this approach (Fig 5A).

When the CpG content of the 10 putative ZAP-dependent targets (“ZAP-DEP,” identified in Fig 5A) was examined, all were relatively CpG rich, with all 10 transcripts exhibiting a CpG frequency greater than the median of all transcripts (Fig 5C, S9 Fig). Moreover, the putative ZAP targets also possessed a substantial number of evolutionarily conserved CpGs (Fig 5D). We hypothesised that downregulation of the putative ZAP targets contributes to the antiviral state. We therefore examined their ability to promote virus replication. Each IRG was exogenously expressed, and their ability to promote the replication of a panel of 14 viruses was considered (Fig 5E). Although some relatively weak enhancement of virus replication was observed in specific instances (Fig 5E), IFN still potently inhibited *Indiana vesiculovirus* (formerly known as vesicular stomatitis virus [VSV]) infection when ZAP was depleted (Fig 5F and 5G). Thus, the ZAP-mediated downregulation of host transcripts does not appear critical for a functional “anti-VSV state.” However, the importance of an IRG to virus replication may not be revealed through exogenous expression, and many more viruses would need to be considered in the absence of IRG expression (i.e., IRG KO) before a key role for IRGs in the “antiviral state” can be excluded.

To validate the putative ZAP targets, we examined the ability of exogenous ZAP expression to decrease the abundance of these specific transcripts. We used the ZAP-S isoform, as this isoform is preferentially upregulated by type I IFNs (Fig 5B) [35]. The shorter ZAP-S mRNA utilises the intron between exons 9 and 10 as a 3′ UTR and does not encode the PARP domain [34,36]. We used a lentiviral vector to express a CRISPR-resistant ZAP-S in A549 ZAP KO guide 1 clone 2 cells (Figs 4C and 5H). We then used RNA-seq to compare the transcriptome of cells expressing exogenous ZAP to control cells transduced with an empty vector. When ZAP-S was expressed (Fig 5H), similar to IFN treatment, the mRNAs that were reduced in abundance were relatively CpG rich (Fig 5I). Moreover, the majority of these downmodulated transcripts have been identified as IRGs in multiple transcriptomic studies (S9 Fig). Accordingly, these putative ZAP targets were predicted to have an increased probability of being IRGs (Fig 5J) using the relevant model from Fig 1. Crucially, as well as reducing the expression of a large number of relatively CpG-rich transcripts, the majority (7/10) of the putative ZAP targets identified in Fig 5A were also significantly downregulated by exogenous ZAP-S (Fig 5K). Although modest in some instances, the observed magnitude of downregulation was similar to the level of repression following IFN treatment (Fig 5A and 5B, S9 Fig). The CpG composition of the 3 IRG transcripts whose abundance did not decrease in the presence of exogenous ZAP-S was similar to the 7 genes whose transcript abundance was reduced by exogenous ZAP (Fig 5L and 5M), suggesting that the overall CpG composition is not the sole determinant of sensitivity to ZAP-S.

When the loss of IRG repression (following ZAP KO) and the reduced IRG transcript abundance (following exogenous ZAP-S expression) are considered together, these data strongly suggest that ZAP is mechanistically responsible for the repression and/or regulation of a subset of IRGs.

## Discussion

The hypothesis that the recognition of nonself influences the composition of innate immune genes was proposed in 2009 [28]. Following the identification of ZAP as a CpG-targeting antiviral effector by Takata and colleagues [10], Stephen Goff commented that “the most far-reaching suggestion arising from this study [Takata et al.] is that ZAP constitutes a factor in the evolution of low CG content in the DNA of host cells” [21]. Given that CpG-targeting antiviral surveillance/effector mechanisms are likely to be at their most active during the IFN-induced antiviral state, we reasoned that the most extreme evolutionary pressures (that could select for biased nucleotide composition) would be exerted on IFN-regulated transcripts. Consistent with these hypotheses, a supervised machine learning approach identified that strongly upregulated ISGs tended to be notably more CpG suppressed than the average gene. Moreover, we demonstrate that IRGs tend to be relatively rich in CpGs and that IFN-stimulated CpG-targeting antiviral defenses downmodulate many CpG-rich host transcripts, leading to their classification as IRGs. Specifically, ZAP KO led to a considerable reduction in the total number of IRGs, suggesting a direct involvement in the suppression of a sizable subset of IRGs. Considering the significant CpG bias present in the interferome, together with the evidence that CpG-targeting effectors modulate gene expression, it seems plausible that IFN-stimulated antiviral effectors have selected for bias in the composition of the interferome. Thus, it is likely that antiviral effectors have influenced the genome composition of both virus and host. Interestingly, CpG composition was not the only discriminatory feature identified using the machine learning approach, and more work is required to understand the selective pressures that may have selected for other compositional biases in the interferome. Notably, the UpA dinucleotide (which is also underrepresented in vertebrate genomes and in viruses with vertebrate hosts) was surprisingly unimportant for distinguishing ISGs from the other gene classes, suggesting that the underrepresentation of UpAs in viral genomes might not be due to the IFN-induced “antiviral state.” Alternatively, the resistance of ISGs to degradation via antiviral pathways, such as the OAS-RNaseL system [37,38], may be achieved by a mechanism that does not require the depletion of UpA and UpU dinucleotides.

Strong selection for high IFN stimulation (or maintenance of efficient IFN expression in the face of autocrine stimulation) could plausibly select against the presence of targeted CpGs. Over evolutionary timescales, the stepwise elimination of targeted CpGs would leave these genes in a highly CpG-suppressed space. Accordingly, we observed lower levels of CpG conservation in IFN genes and ISGs than in IRGs. Moreover, the biased CpG content is an ancient property of the interferome that we observed in multiple vertebrates, some of whom have been separated by over 300 million years of evolution. Although limiting the analysis to the 50 most DE genes was arbitrary, the compositional bias was only easily observed at the extremes of the interferome. We believe that the visualisation of the same trend in multiple species supports the relevance of this approach. Importantly, this trend was not due to repeated sampling of the same genes, as the bias remained constant even though more than 600 different orthologous clusters are represented in Figs 1 and 2.

As a word of caution, it is difficult to use contemporary observations to ascribe causation to the selection of genomic signatures that have accumulated over deep timescales. We are unable to rule out that other processes, such as reduced CpG methylation within the genomic loci of highly expressed ISGs (which could be more conducive to rapid induction of gene expression), have selected for low CpG composition in ISGs. Thus, although self-targeting represents a plausible and parsimonious explanation that links our observed transcriptomic changes to the observed compositional biases, this link is currently only correlative.

Based on our RNA-seq data, ZAP targeting of host mRNAs appears to be mechanistically responsible for the IFN-induced repression of multiple IRGs. Whether ZAP-mediated mRNA degradation represents “collateral damage” (i.e., a tolerated consequence of the antiviral state), or whether specific transcripts have been selected to be regulated by ZAP (i.e., ZAP-targeted repression of specific transcripts enhances immune responses), is not yet fully resolved. It is worth noting some key differences between these gene classes. Many ISGs are expressed at low or undetectable levels prior to IFN induction. This means that following IFN stimulation, an increase in transcript abundance can correspond to remarkable fold changes (sometimes >1,000-fold). This “zero-to-hero” lifestyle provides added consistency/robustness to RNA-seq studies of ISGs as this class is relatively indifferent to the cell type or cellular state prior to IFN stimulation. In contrast, IRGs, by definition, must be robustly expressed prior to IFN stimulation, and IRGs are seldom repressed more than 2-fold. This technical difference contributes to making IRGs a more variable class of genes. Moreover, many individual ISGs are conserved over large phylogenetic distances and are consistently upregulated in divergent cell lineages [2,4,39], while this is usually more variable for IRGs [2,4]. Crucially, although the identity of the individual transcripts that are repressed is variable, the compositional characteristics of IRGs appear relatively constant. Thus, we believe collateral damage (as opposed to regulation) underlies the downregulation of most IRGs. A collateral damage hypothesis is also consistent with the reduced ability of our machine learning classifier to robustly distinguish IRGs from random genes. In contrast, the consistent downregulation of a small number of IRGs implies a regulatory role. We thus speculate that ZAP-mediated regulation of specific IRGs occurs within the broader context of “off-target” downregulation of many relatively CpG rich “bystander” mRNAs (not necessarily related to the antiviral state).

While the biological consequences of the regulation imposed by ZAP targeting are largely uncharacterised, it is logical to predict that they will be aligned with the previously defined functions of the interferome. For example, a proapoptotic programme is an ancient function of the interferome [4,40]. Thus, ZAP-mediated downregulation of TNFRSF10D and CAMKK1, both of which have been ascribed antiapoptotic functions [33,41,42], would likely potentiate the proapoptotic effect of IFNs. Moreover, the ability to directly interfere with viral replication is a key function of the interferome. As both CAMKK1 and KDM6B are necessary for efficient herpesvirus replication or reactivation [43,44], the reduced levels of these gene products could potentially inhibit viral replication. In support of this notion, ablation of CAMKK1 expression has recently been shown to inhibit cytomegalovirus replication in vitro [45]. In contrast, the observed ability of IFN to inhibit *Indiana vesiculovirus* (VSV) infection in the absence of ZAP indicates that ZAP-targeted IRGs are not essential for a functional antiviral state. Importantly, redundancy in the antiviral state means that the removal of a single antiviral pathway does not always result in a rescue of infection or replication. Notably, VSV (like many viruses) is sensitive to multiple antiviral ISGs [46–48]. Thus, much more work will be required to determine the contribution (if any) that IRGs might make to the inhibition of specific viruses.

Although the most DE IRGs tended to be relatively CpG rich, we observed no absolute relationship between the CpG content of a transcript and whether ZAP was required for its repression. Clearly, not all CpGs are targeted equally by ZAP, and it is likely that RNA structure/context is crucial for CpG-targeted degradation [17,19,21,49]. Thus, the tendency for ZAP to target relatively CpG-rich IRGs likely represents the increased probability that these IRGs have the “right kind” of CpGs.

Interestingly, ZAP-independent IRGs also tended to be enriched in CpGs. Moreover, using a supervised machine learning approach, ZAP-dependent and ZAP-independent IRGs were compositionally indistinguishable. Thus, we are currently unable to predict which CpGs in host transcripts might be recognised as pathogen-associated molecular patterns (PAMPs). Given the similarity between ZAP-dependent and ZAP-independent IRGs, it is tempting to speculate that other IFN-stimulated CpG-targeting antiviral effectors might also exist, or that the ZAP-mediated regulation of IRGs is redundant. Importantly, other CpG-targeting effectors could also select for compositional biases in host transcripts, and the relative contribution of ZAP may only become apparent once the context of ZAP-mediated CpG recognition is fully mechanistically understood. Therefore, examining whether all synthetically deoptimised, CpG-rich viruses are rescued by ZAP depletion is likely to be an important avenue of future research.

Whether ZAP influences the expression of IFN genes directly has not been tested here. Intriguingly, it has been proposed that ZAP-S might regulate the expression of human IFN genes [36]. This study focused on the relatively CpG rich type III IFN genes, although the authors also proposed that type I IFNs could be regulated by ZAP [36]. Thus, it is possible that CpG-targeting effectors might also have influenced the CpG composition of IFN genes, although this does not appear to be the case in all the species examined. Although extreme CpG suppression is observed in the type I IFN genes of humans and some other mammals, this trend appears to be more evolutionarily variable than the bias in the composition of the interferome. One potential explanation for this could be that IFNs transiently trigger the antiviral state, and, therefore, might not need to be expressed for long periods in the face of autocrine signalling (and may even have been selected as targets in some species). Conversely, ISG transcripts must persist in the IFN-stimulated cell to potentiate the antiviral state. More studies will be needed to detail how/whether all IFN transcripts escape inhibition by the antiviral state and how these processes might vary between species. Similarly, although the CpG bias in the interferome was observed in divergent animal species, the horse was a notable exception to this trend. Both equine ISGs and type I IFNs were not strongly CpG suppressed and exhibited levels of CpG suppression that were very similar to the equine transcriptome as a whole. Notably, we could not detect any obvious features at the horse ZC3HAV1 locus that suggested equine ZAP might be inactive in horses. However, we have equally been unable to definitively support or refute the existence of ZAP-S in horses (although this was the case for multiple species under consideration in this study). Thus, further studies are required to investigate whether CpG-targeting effectors are active in equine cells. Conversely, we currently have no explanation for the more pronounced CpG bias observed in the ovine and canine interferomes, and more work is required to investigate whether CpG-based pattern recognition is particularly effective in these species.

Viral RNAs are simultaneously under surveillance from a multitude of host defenses and sensors. Because these highly upregulated ISGs must be efficiently expressed in the face of all these defenses, it is likely that ISGs and viruses will share multiple compositional characteristics (to facilitate efficient gene expression during an active antiviral state). The influence of self versus nonself discrimination on the composition of host genomes may therefore be an underappreciated evolutionary process. Once a nucleic acid–based pattern recognition system emerges, the removal of said patterns will be selected for in both host and pathogen. Thus, the observed underrepresentation of features exploited for virus recognition (in contemporary host genomes) may have become exaggerated over evolutionary timescales and become particularly exaggerated in ISG transcripts. Identifying the shared features of viral RNAs and highly upregulated ISGs could, in the future, reveal novel mechanisms used by the host to sense and destroy invading pathogens, help identify viral reservoirs, and improve vaccine strategies.

## Materials and methods

### Determining dinucleotide frequencies

A database of CpG dinucleotide frequencies was prepared for 10 species for which ISG/IRG data were available [4] (http://isg.data.cvr.ac.uk). Briefly, for all species, all cDNAs and CDSs were downloaded from Ensembl via their FTP website (release 92: ftp://ftp.ensembl.org/pub/release-92/fasta), except the 100 mouse genes, which were analysed at a later date using Ensembl release 99. For each sequence, both the relative frequency of CpGs and the observed/expected ratio (normalised CpG frequency) were calculated using the following formulas. The CpG dinucleotide frequency (CpG frequency) was counted for each transcript, giving a raw count that was then normalised by cDNA length. Thus, CpG frequency = #CG dinucleotides/(length-1). A second value normalising for GC richness was also calculated, where CpG frequency (normalised) = CpG frequency/ (proportion C * proportion G), equivalent to observed/expected or odds ratio. Where observed CpG and expected CpG are plotted, the observed = #CG dinucleotides, while the expected CpG = Prop(C)*Prop(G)*(Length of transcript-1), where Prop(C) and Prop(G) are the proportions of C and G nucleotides in the transcript of that specific gene. These databases were then matched against ISGs/IRGs according to Ensembl gene ID. The analysis of the CpG composition of human cDNAs was also conducted using cDNA sequences from Ensembl (ftp://ftp.ensembl.org/pub/release-92/fasta/homo_sapiens/). The location of each transcript was mapped, and redundant transcripts for each gene were removed, retaining the longest transcript variant of each gene. In multiple panels, we compared the composition of the 50 most DE ISGs and IRGs. We opted to compare the top 50 most DE ISGs and IRGs based upon S1 Fig. Due to the non-normal distribution of CpG frequencies, the nonparametric Wilcoxon rank sum test was used for comparing independent groups of genes. Similarly, where multiple groups were compared, we used a Kruskal–Wallis rank sum test followed by post hoc analysis using the Dunn test including Benjamini–Hochberg correction for multiple comparisons. The frequency of each dinucleotide was calculated for the top 50 human ISGs and IRGs, and the normalised dinucleotide frequency was used (equivalent to the observed/expected or odds ratio). Each set of 50 random genes was selected to be representative of genes unaffected by IFN treatment [4]. First, genes DE at false discovery rate (FDR) <0.05 were subtracted from the remainder of the genome. Non-DE genes were further subsetted to leave only those for which there was evidence of transcription in every sample [4]. Finally, 50 random genes were selected using the sample function in R.

### Feature-based machine learning

To gauge the relative importance of various compositional features, 3 classifiers were trained to distinguish (1) the top 50 ISGs from the top 50 IRGs of each species; (2) the top 50 ISGs from 50 randomly selected genes from each species; and (3) the top 50 IRGs from the same set of 50 randomly selected genes (ISGs and IRGs were ranked by mean Log_2_FC (fold change), whereas random genes (see above) were randomly selected from the remaining transcribed genes that were not DE in response to IFN). For one spliced/mature transcript representing each gene, 35 features were calculated across the entire sequence describing GC content, AT content, dinucleotide biases (*N* = 16; calculated as described previously [50]), and the proportion of bases dedicated to each dinucleotide (dinucleotide proportion, *N* = 16). An additional 133 features were calculated to specifically describe the open reading frames of all protein CDSs, including open reading frame–specific versions of GC content, AT content, dinucleotide biases (*N* = 16), and dinucleotide proportion (*N* = 16), along with codon usage biases (*N* = 64), amino acid biases (*N* = 21), and position-specific dinucleotide biases at either codon bridge (*N* = 16) or non-bridge positions (*N* = 16) [50].

These features were used to train 3 independent boosted regression tree classifiers using the DART algorithm of the XGBoost library, combining genes from all species into a single dataset [51,52]. A search for the optimal combination of tuning parameters was performed using the caret library in R, searching across 100 randomly selected parameter combinations [53,54]. Models were evaluated by randomly splitting the data into 5 equally sized bins and using each bin, in turn, as a test dataset to score the prediction accuracy of a model trained on the remaining 4 bins (i.e., 5-fold cross-validation). This procedure was repeated 50 times to assess the variation in predictive accuracy before training the final model using all available data.

The relative importance of the different genome features in the classification of individual genes was assessed in this final model by calculating approximate Shapley values using the TreeSHAP algorithm [23,24]. To obtain a single value for each broad feature class, often consisting of highly correlated features, Shapley values were summed across all features making up the broader feature class. For example, the total importance of ApU dinucleotides was calculated as the sum of mean absolute Shapley values for ApU bias across the entire gene, ApU proportion across the entire gene, ApU bias in open reading frames, ApU proportion in open reading frames, ApU bias at codon bridge positions, and ApU bias at non-bridge positions. Finally, the importance of each feature class was calculated as the mean of absolute Shapley values across all genes in the data. To allow comparison across independent classifiers, values were rescaled to lie between 0 and 1.

### GOrilla analysis and immune gene selection

Enrichment of GO terms was assessed using GOrilla [27]. Lists of Ensembl gene IDs, ranked according to Log_2_FC or CpG content, as appropriate, were used as inputs. Transcripts shorter than 100 nucleotides in length were excluded from these analyses. Default parameters were then used to generate lists of enriched terms under “Process” and “function.” Genes were classified as either immune genes, ISGs/IRGs, or IFNs using the ImmPort database [29], previously published transcriptomics analyses [4] or text mining gene names containing “IFN,” respectively.

### Cells and gene editing

Human A549 cells were cultured in Dulbecco’s Modified Eagle Medium (DMEM) with 9% fetal calf serum (FCS) supplemented with gentamicin. Gene editing was achieved using the lentiGuide-Puro system in accordance with the Zhang Lab protocols [55]. Oligos specifying the following ZAP target sequences were used: 5′- ATGTGGAGTCTTGAACACGG-3′ (1), 5′- GCAACTATTCGCAGTCCGAG-3′ (2), and 5′-ACTCTCTGGACTGAACAAAG-3′ (5) to generate single-guide RNA (sgRNA) encoding vectors. A549 cells were either transduced with vectors encoding Cas-9 and the relevant ZAP-targeting sgRNAs or transduced in parallel with vectors encoding Cas-9 and no sgRNA (no guide). To maximise the frequency of gene disruption, the bulk KO cells examined in Fig 3 were transduced with a mixture of both sgRNA 1 and sgRNA 5 vectors. Single cell clones were generated using limiting dilution.

### RNA-seq

For the majority of experiments, we selected a sample size of 3 independent replicates (3 separate cultures per condition) as an optimal balance between experimental power and the resources available. The exception to this was the transcriptomic analysis of ZAP KO clones, where 5 control single cell clones were compared to 5 ZAP KO single cell clones, with each clone treated as a replicate. This experiment was initially designed to compare 6 clones v 6 clones. However, following principle component analysis, one ZAP KO clone was an extreme outlier (guide 2 clone 3). Thus, the guide 2 clone 3 and a randomly selected control clone (No guide clone 11) were discarded from all subsequent analyses.

Cells were treated or untreated with 1,000 units/ml IFNβ (PBL Assay Science, New Jersey, USA) for 4 hours prior to RNA extraction (RNeasy, Qiagen, Hilden Germany). Poly(A)-enriched Illumina RNA-seq libraries were prepared and run. Briefly, RNA concentration and integrity were determined using a Qubit Fluorimeter (Life Technologies, California, USA) and 4200 TapeStation (Agilent, California, USA). All samples had an RNA integrity number of 8 or above. Moreover, 500 ng of total RNA was used to prepare libraries for sequencing, using an Illumina TruSeq Stranded mRNA HT kit (Illumina, California, USA), according to the manufacturer’s instructions. Libraries were pooled in equimolar concentrations and sequenced using an Illumina NextSeq 500 sequencer (high output cartridge), generating single end reads with a length of 75 bp. The raw FASTQ read output was first assessed using FASTQC. Sequencing adapter sequences were removed from reads using TrimGalore prior to mapping against the human genome (GRCh38.p12) using HISAT2. Mapped reads aligning to genes annotated in a .gtf file of the human genome were counted using HTseq-Count. Reads mapping at a frequency of <1 in greater than one half of the samples were removed prior to differential expression analysis using edgeR. To correct for multiple testing in these analyses, we applied the Benjamini–Hochberg correction to generate an FDR value for each gene. Genes DE with an FDR <0.05 were considered significant. The raw FASTQ files generated during this project have been submitted to the European Bioinformatics Institute (EBI) under project accession numbers PRJEB29677 and PRJEB39825.

### Exogenous ZAP expression

Three independent cultures of A549 ZAP KO cells (ZAP guide 1 clone 2) were transduced with lentiviral SCRPSY vectors expressing either ZAP-S or an empty backbone as control. Cells were transduced in the presence of polybrene (final concentration: 8 μg/ml) and spinoculated at 400 × g for 1 hour at room temperature. After 48 hours, cells were harvested in TRIzol (Ambion, Thermo Fisher Scientific, Texas, USA) prior to RNA extraction (RNeasy, Qiagen) and RNA-seq analysis, as described above. Additional samples were harvested for FACS analysis to confirm the percentage of transduction (EMPTY 92.2% and ZAP-S 76%) alongside samples for western blot analysis.

### Viruses and titrations

Human A549 cells and derivatives were stimulated with 1,000 units of IFNβ (PBL Assay Science) for 24 hours before being infected with a serially diluted challenge of a single cycle *Indiana vesiculovirus* system, rVSVΔG-GFP [56]. At 16 hours postinfection, the levels of infection were quantified using flow cytometry.

Human MT4 cells were transduced with IRG-encoding SCRPSY lentiviral vectors [57] and 48 hours later were infected with 7-point serially diluted doses of a panel of GFP-encoding viruses. Infected cells were enumerated using flow cytometry and the titre of each virus (in cells transduced with each IRG) was extrapolated from 3 points in the linear range. Each titre is plotted as a fold change in titre (relative to cells transduced with the empty vector). The virus panel consisted of HIV-1 (NHG, JQ585717.1), human alphaherpesvirus 1 (HSV-1) [58], adenovirus 5 [59], *Indiana vesiculovirus* [56], and *Chandipura vesiculovirus* [60]; *Bunyamwera orthobunyavirus* and *Rift Valley fever phlebovirus* (RVFV 35/74) [61]; and parainfluenza virus 3, parainfluenza virus 5, Sendai virus (ViraTree), and *Semliki Forest Virus* [62]. In addition, the design and rescue of the NS1-eGFP expressing A/Puerto Rico/8/1934 (H1N1) virus has been described previously [47]. The A/California/04-061-MA/2009(H1N1) NS1-eGFP virus was rescued by replacing the parental NS segment (GenBank accession KX134783.1) with the PR8 NS1-eGFP segment. An additional NS1-eGFP expressing NS segment was designed using the NS1 and NEP sequences of A/mallard/Netherlands/10-Cam/1999(H1N1) (GenBank accession KC209519.1) and used to rescue the Mallard-GFP virus in the same fashion as the PR8 NS1-eGFP virus described previously [47]. The IRG-encoding vectors were synthesised (GENEWIZ, New Jersey, USA), and the following sequences were used: NM_003840 (TNFRSF10D), NM_001947 (DUSP7), NM_032294 (CAMKK1), NM_022766 (CERK), NM_134269 (SMTN), NM_001037125 (UNKL), NM_145290 (ADGRA3), NM_001010924 (FAM171A1), NM_001080424 (KDM6B), and NR_024031 (DANCR, note that DANCR is not protein coding).

### Western blotting

Western blotting was carried out as described previously [63] using a Li-COR Odyssey scanner and the following antibodies: ZAP (Proteintech (Illinois, USA) 16820-1-AP), Phospho-STAT1, (Cell Signaling Technology 9167), GAPDH, (Cell Signaling Technology (Massachusetts, USA) #2118), FAM171A1 (Abcam (Cambridge, UK) ab229247), or actin (JLA20 hybridoma, courtesy of the Developmental Studies Hybridoma Bank, University of Iowa). In Fig 5G, proteins were visualised using an ECL system (GE Healthcare (Illinois, USA) RPN2106) and the above antibodies.

### CpG conservation

Alignments available for the 50 most DE IRGs (*n* = 34), ISGs (*n* = 30), and type I IFNs (*n* = 4) were obtained from existing primate alignments [31] and used to estimate the degree of CpG conservation. Each alignment includes 9 primate species: *Callithrix jacchus*, *Chlorocebus sabaeus*, *Gorilla gorilla*, *Homo sapiens*, *Macaca mulatta*, *Nomascus leucogenys*, *Pan troglodytes*, *Papio anubis*, and *Pongo abelii*. For each site in the alignment that had at least 1 CpG, the percentage of CpG conservation was computed by normalising the number of CpGs by the total number of sequences in the alignment. CpG/kb was then determined from the ratio of the number of CpGs at each degree of conservation to the total gene length of all the sequences.

### Codon Adaptation Index

The Codon Adaptation Index (CAI) for each CDS in the genome was calculated using an established method [25]. Briefly, a relative adaptiveness weight [*w*] for each codon [*c*] is calculated using the frequency of the codon [*f*_*c*_] and the frequency of the most frequent synonymous codon for the corresponding amino acid [max(*f*_*c*_*)*] across all CDSs in the genome:

$$w_{c}=\frac{f_{c}}{max(f_{c})}$$

The CAI of each individual CDS is then calculated as the geometric mean of the weight of each codon in the sequence over its codon length [*L*]:

$$CAI=\frac{\prod_{c=1}^{L}w_{c}}{L}$$

### Data retrieval from the “Interferome” database

To visualise the expression levels of genes upon IFN treatment in other datasets, the Interferome database was used. Data from the Interferome v2.01 [2] were downloaded from the web application http://www.interferome.org/. The database was searched for a list of Ensembl IDs, and the following additional search criteria were used: Interferon Type I, Species *Homo Sapiens*, Fold Change Up/Down 1.0. The retrieved experimental data of those genes was downloaded as a text file and used for downstream analysis.

## Supporting information

S1 Fig Sliding window analysis of the CpG composition of ISGs and IRGs. **(A)** The CpG frequency (upper) and normalised (to GC content) frequency (lower) in transcripts from primary human fibroblasts DE in response to type I IFN. CpG values were extracted for each human ISG and IRG defined in [4]. **(B)** The median CpG frequency (left) and normalised frequency (right) of windows of 50 genes. ISGs and IRGs were ranked according to fold change (FC) and the average CpG value calculated for a window of 50 genes. Window medians were calculated at 10-gene intervals. **(C)** CpG frequency (upper panels) and normalised frequency (lower panels) distributions for the 50-gene windows defined in (B). Each distribution is compared to the same 50 human genes selected at random. Statistical significance between ISG and IRG distributions of CpG frequencies was assessed by Wilcoxon rank sum test. The horizontal dotted line in each plot represents the median value for all transcripts in the human genome. The underlying data from this figure are openly available (http://dx.doi.org/10.5525/gla.researchdata.1159). DE, differentially expressed; GC, guanine–cytosine; IFN, interferon; IRG, interferon-repressed gene; ISG, interferon-stimulated gene.
(TIF)

S2 Fig Normalised dinucleotide composition of the most DE ISGs and IRGs. The 50 most DE human ISGs and IRGs (ranked by Log_2_FC) were selected and the frequency of each dinucleotide calculated. Each row represents the first nucleotide and each column the second nucleotide. The median frequency of each dinucleotide in the human transcriptome is indicated by the horizontal dotted line. Statistical significance between ISG and IRG distributions of dinucleotide frequencies was assessed by Wilcoxon rank sum test. The underlying data from this figure are openly available (http://dx.doi.org/10.5525/gla.researchdata.1159). DE, differentially expressed; IRG, interferon-repressed gene; ISG, interferon-stimulated gene.
(TIF)

S3 Fig The relative importance of features used to distinguish IRGs from random genes. The relative discriminating power of the most significant features identified using a supervised machine learning approach. Features are ranked according to their ability to discriminate the 50 most DE IRGs from 50 non-DE transcripts selected randomly from the remainder of the transcribed human genome. The underlying data from this figure are openly available (http://dx.doi.org/10.5525/gla.researchdata.1159). DE, differentially expressed; IRG, interferon-repressed gene.
(TIF)

S4 Fig The vertebrate interferome has a CpG bias. **(A)** The CpG values normalised for both length and GC content of the top 50 most DE human ISGs and IRGs (ranked by Log_2_FC). The dotted line represents the median frequency of all transcripts in the relevant genome. A random sample of non-DE genes is included for reference. Bar and whiskers represent the median and interquartile range of each distribution, respectively. **(B)** The remaining 9 vertebrate species plotted as in (A). The underlying RNA-seq data used were previously published open-access data [4] and were also described in the Fig 1 legend. Significance was determined using the Wilcoxon rank sum test with continuity correction. The underlying data from this figure are openly available (http://dx.doi.org/10.5525/gla.researchdata.1159). DE, differentially expressed; GC, guanine–cytosine; IRG, interferon-repressed gene; ISG, interferon-stimulated gene; RNA-seq, RNA sequencing.
(TIF)

S5 Fig CpG suppression in human transcripts and GO analysis of the most suppressed human transcripts. **(A)** Observed over expected CpG values (equivalent to CpG frequencies normalised for GC content) for every transcript in the human genome. The diagonal dotted line represents equal observed and expected CpG values (3 cDNAs were excluded from the plot due to excessive length but did not display different trends). **(B)** Significant GO terms associated with 1,000 human transcripts over 100 nucleotides in length with the lowest CpG content (normalised for GC composition). Blue bars indicate GO processes and functions associated with the immune response. Removal of IFNα2 and IFNα14 ablated the significance of these terms. * Full GO process names: “positive regulation of peptidyl-serine phosphorylation of STAT protein” and “negative regulation of adaptive immune response based on somatic recombination of immune receptors built from immunoglobulin superfamily domains”. The underlying data from this figure are openly available (http://dx.doi.org/10.5525/gla.researchdata.1159). GC, guanine–cytosine; GO, gene ontology; IFN, interferon.
(TIF)

S6 Fig Statistical analysis of Fig 3B. Matrices highlighting the significance of potential comparisons in Fig 3B. Significance was assessed using the Kruskal–Wallis rank sum test followed by post hoc analysis using the Dunn test including Benjamini–Hochberg correction for multiple comparisons. The underlying data from this figure are openly available (http://dx.doi.org/10.5525/gla.researchdata.1159).
(TIF)

S7 Fig CpG composition of type I IFN genes. **(A)** The CpG frequency and (B) the CpG frequency normalised for GC composition of type I IFN genes relative to 50 genes selected at random from the remainder of the genome. The dotted line represents the median CpG frequency of all transcripts in the relevant genome. Statistical significance between IFN gene and random gene distributions of CpG frequencies was assessed by Wilcoxon rank sum test. The underlying data from this figure are openly available (http://dx.doi.org/10.5525/gla.researchdata.1159). GC, guanine–cytosine; IFN, interferon.
(TIF)

S8 Fig Selecting IRGs for further investigation. Venn diagrams representing the numbers of **(A)** significantly DE ISGs or **(B**) significantly DE IRGs from the RNA-seq of A549 cells from either (“bulk”) or (“clones”) stimulated with 1,000 units/ml of IFNβ (4 hours). To increase confidence in the classification of these DE genes, in all cases, these data were filtered to only consider genes that were significantly DE in either bulk populations or clonal lines and were independently classified as DE in primary fibroblasts [4]. Arrows highlight the percentages of genes that differ (no longer DE) between the ZAP KO and “no guide” controls. The 15 IRGs present in bulk and clonal populations in the “no guide” control (left-hand side of panel B) are expanded in Fig 5A. The underlying data from this figure are openly available (http://dx.doi.org/10.5525/gla.researchdata.1159). DE, differentially expressed; IFN, interferon; IRG, interferon-repressed gene; ISG, interferon-stimulated gene; RNA-seq, RNA sequencing; ZAP, zinc-finger antiviral protein.
(TIF)

S9 Fig The putative ZAP targets are documented IRGs. **(A)** A heat map describing the response of the putative ZAP targets to IFN treatment is shown based on previous studies extracted from the Interferome database (www.interferome.org). Colour and intensity reflects the direction and extent of differential expression observed in the individual studies: ISGs are orange, and IRGs are blue. **(B)** As in (A), a heat map is shown summarising the previous measurements in the Interferome database of 46 genes (46 transcripts of the top 50 were retrievable from the database), which are reduced in abundance following exogenous ZAP expression. Putative ZAP targets are highlighted using red typeface. **(C)** As in (A) and (B), a heat map is shown summarising the previous measurements in the Interferome database of 46 genes which are increased in abundance following exogenous ZAP expression. **(D–G)** Matrices highlighting the significance of potential comparisons in Fig 5C (D and E), 5L (F) and 5M (G). Significance was assessed using the Kruskal–Wallis rank sum test followed by post hoc analysis using the Dunn test including Benjamini–Hochberg correction for multiple comparisons. The underlying data from this figure are openly available (http://dx.doi.org/10.5525/gla.researchdata.1159). IFN, interferon; IRG, interferon-repressed gene; ISG, interferon-stimulated gene; ZAP, zinc-finger antiviral protein.
(TIF)

S1 Table The ISGs and IRGs from Fig 2. IRG, interferon-repressed gene; ISG, interferon-stimulated gene.
(XLSX)

S2 Table Details of putative ZAP targets. ZAP, zinc-finger antiviral protein.
(XLSX)

S1 Raw Images Raw images of the WBs presented in this study. The underlying image files are openly available (http://dx.doi.org/10.5525/gla.researchdata.1159). WB, western blot.
(TIF)

## Abbreviations

CAI: Codon Adaptation Index
CDS: coding sequence
DE: differentially expressed
DMEM: Dulbecco’s Modified Eagle Medium
FCS: fetal calf serum
FDR: false discovery rate
GC: guanine–cytosine
GO: gene ontology
HSV-1: human alphaherpesvirus 1
IFN: interferon
IRG: interferon-repressed gene
ISG: interferon-stimulated gene
KO: knockout
PAMP: pathogen-associated molecular pattern
RNA-seq: RNA sequencing
RVFV: Rift Valley fever phlebovirus
sgRNA: single-guide RNA
VSV: vesicular stomatitis virus
ZAP: zinc-finger antiviral protein
