# Supplementary figures and images for: The antiviral state has shaped the CpG composition of the vertebrate interferome to avoid self-targeting
Source: PLoS Biol. 2021 Sep 7;19(9):e3001352. doi: 10.1371/journal.pbio.3001352 (PMC8423302; doi:10.1371/journal.pbio.3001352)

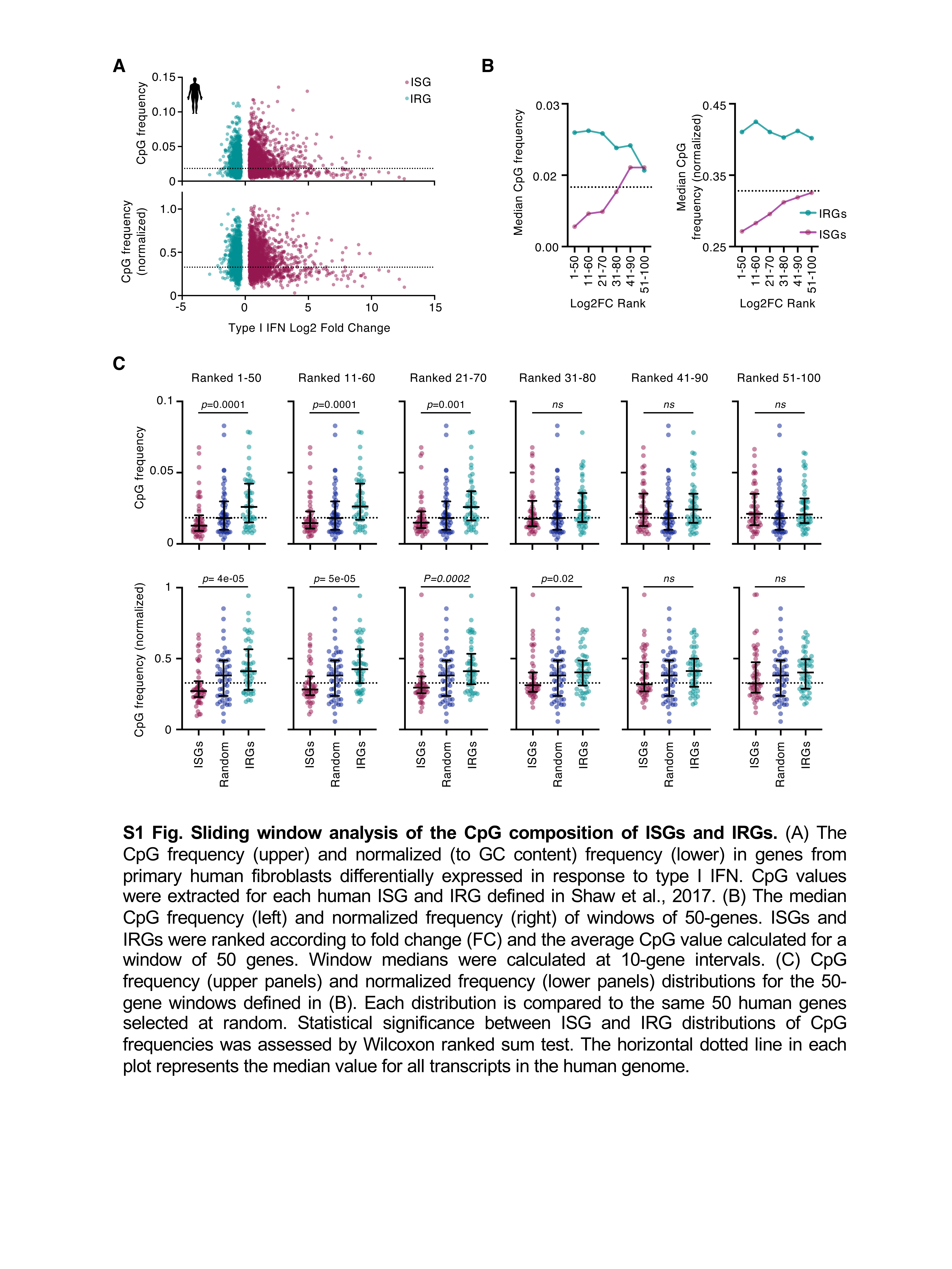

Supplement: S1 Fig — (A) The CpG frequency (upper) and normalised (to GC content) frequency (lower) in transcripts from primary human fibroblasts DE in response to type I IFN. CpG values were extracted for each human ISG and IRG defined in [4]. (B) The median CpG frequency (left) and normalised frequency (right) of windows of 50 genes. ISGs and IRGs were ranked according to fold change (FC) and the average CpG value calculated for a window of 50 genes. Window medians were calculated at 10-gene intervals. (C) CpG frequency (upper panels) and normalised frequency (lower panels) distributions for the 50-gene windows defined in (B). Each distribution is compared to the same 50 human genes selected at random. Statistical significance between ISG and IRG distributions of CpG frequencies was assessed by Wilcoxon rank sum test. The horizontal dotted line in each plot represents the median value for all transcripts in the human genome. The underlying data from this figure are openly available (http://dx.doi.org/10.5525/gla.researchdata.1159). DE, differentially expressed; GC, guanine–cytosine; IFN, interferon; IRG, interferon-repressed gene; ISG, interferon-stimulated gene. (TIF) [file pbio.3001352.s001.tif]

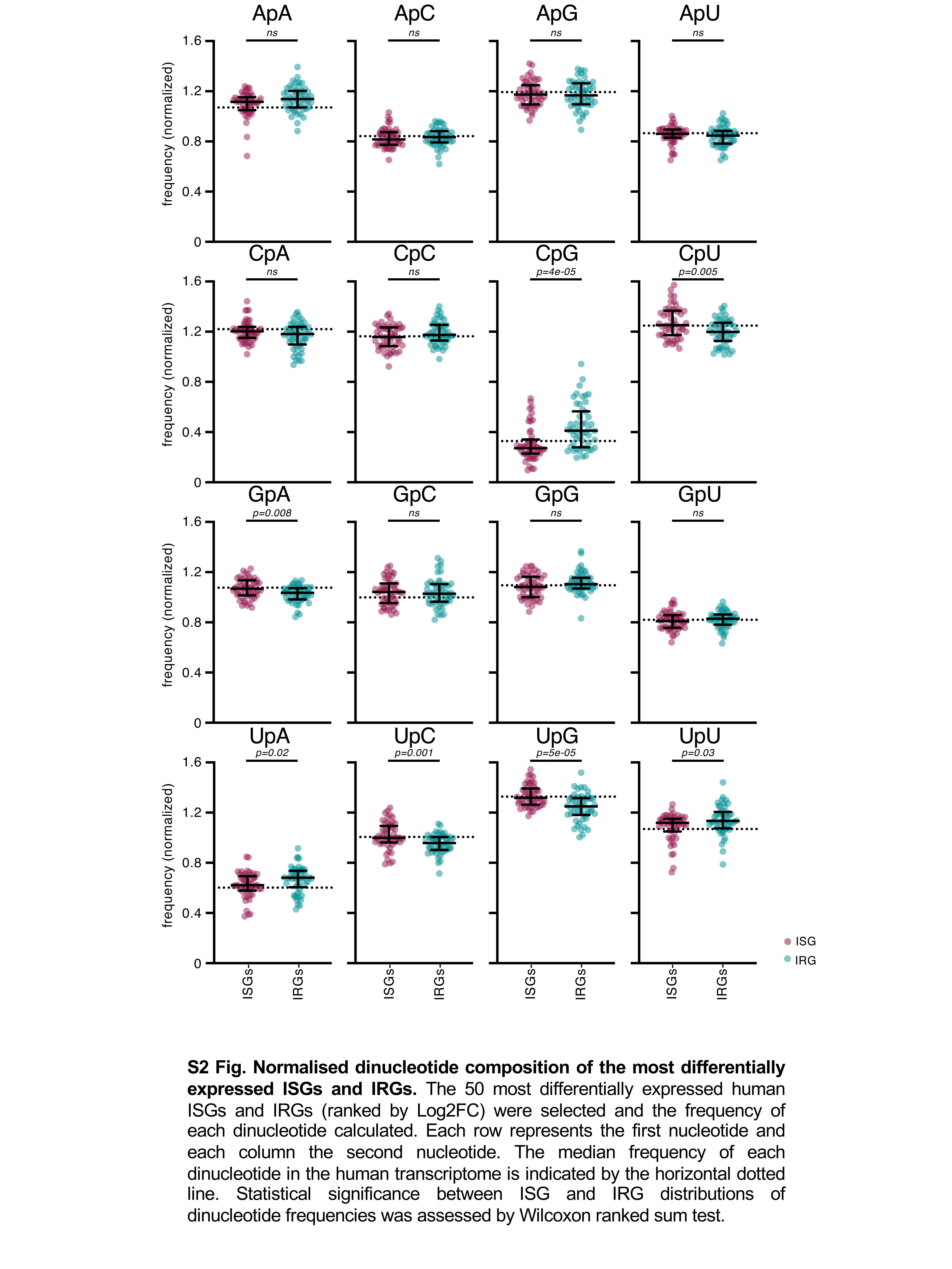

Supplement: S2 Fig — The 50 most DE human ISGs and IRGs (ranked by Log2FC) were selected and the frequency of each dinucleotide calculated. Each row represents the first nucleotide and each column the second nucleotide. The median frequency of each dinucleotide in the human transcriptome is indicated by the horizontal dotted line. Statistical significance between ISG and IRG distributions of dinucleotide frequencies was assessed by Wilcoxon rank sum test. The underlying data from this figure are openly available (http://dx.doi.org/10.5525/gla.researchdata.1159). DE, differentially expressed; IRG, interferon-repressed gene; ISG, interferon-stimulated gene. (TIF) [file pbio.3001352.s002.tif]

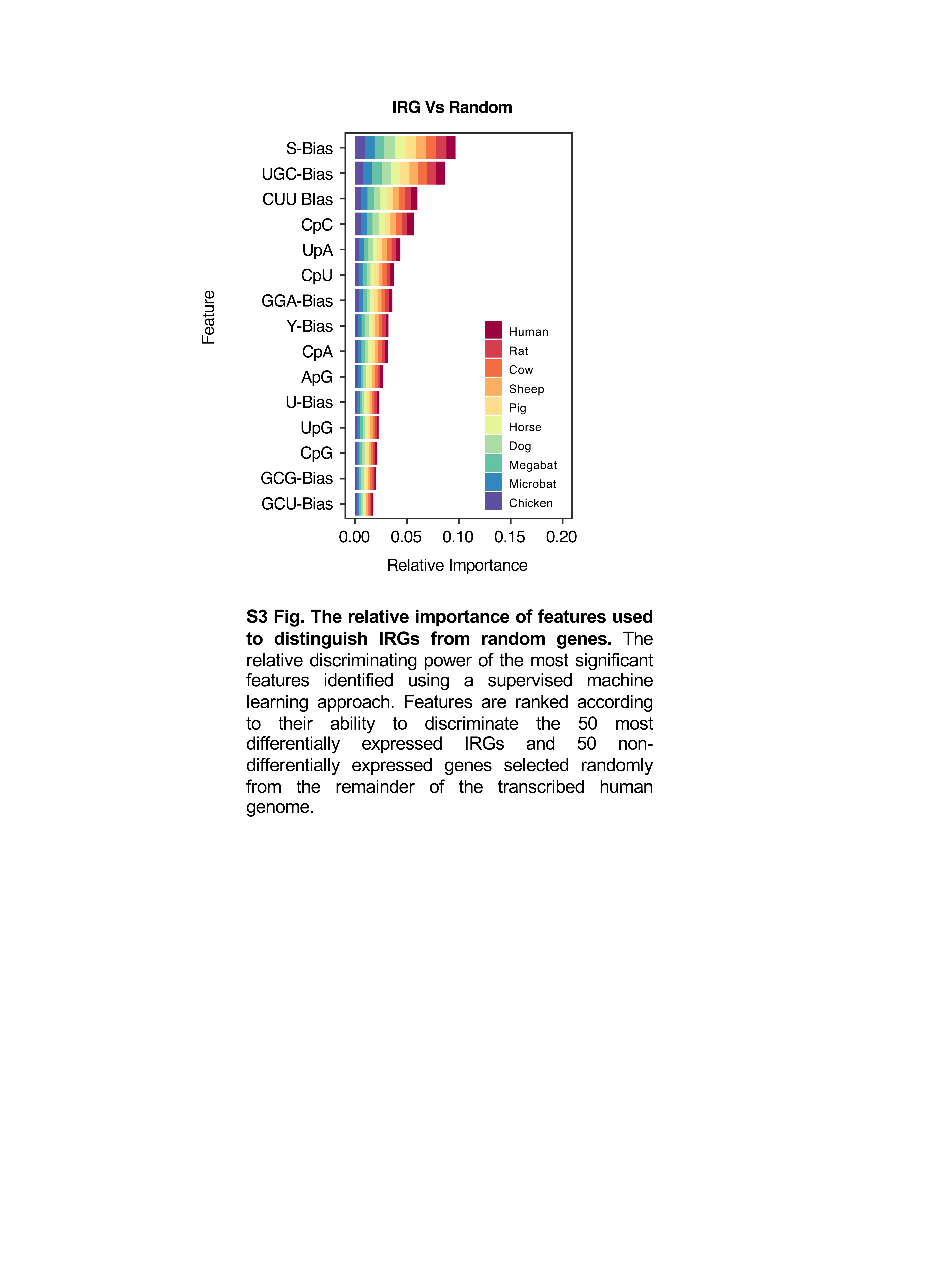

Supplement: S3 Fig — The relative discriminating power of the most significant features identified using a supervised machine learning approach. Features are ranked according to their ability to discriminate the 50 most DE IRGs from 50 non-DE transcripts selected randomly from the remainder of the transcribed human genome. The underlying data from this figure are openly available (http://dx.doi.org/10.5525/gla.researchdata.1159). DE, differentially expressed; IRG, interferon-repressed gene. (TIF) [file pbio.3001352.s003.tif]

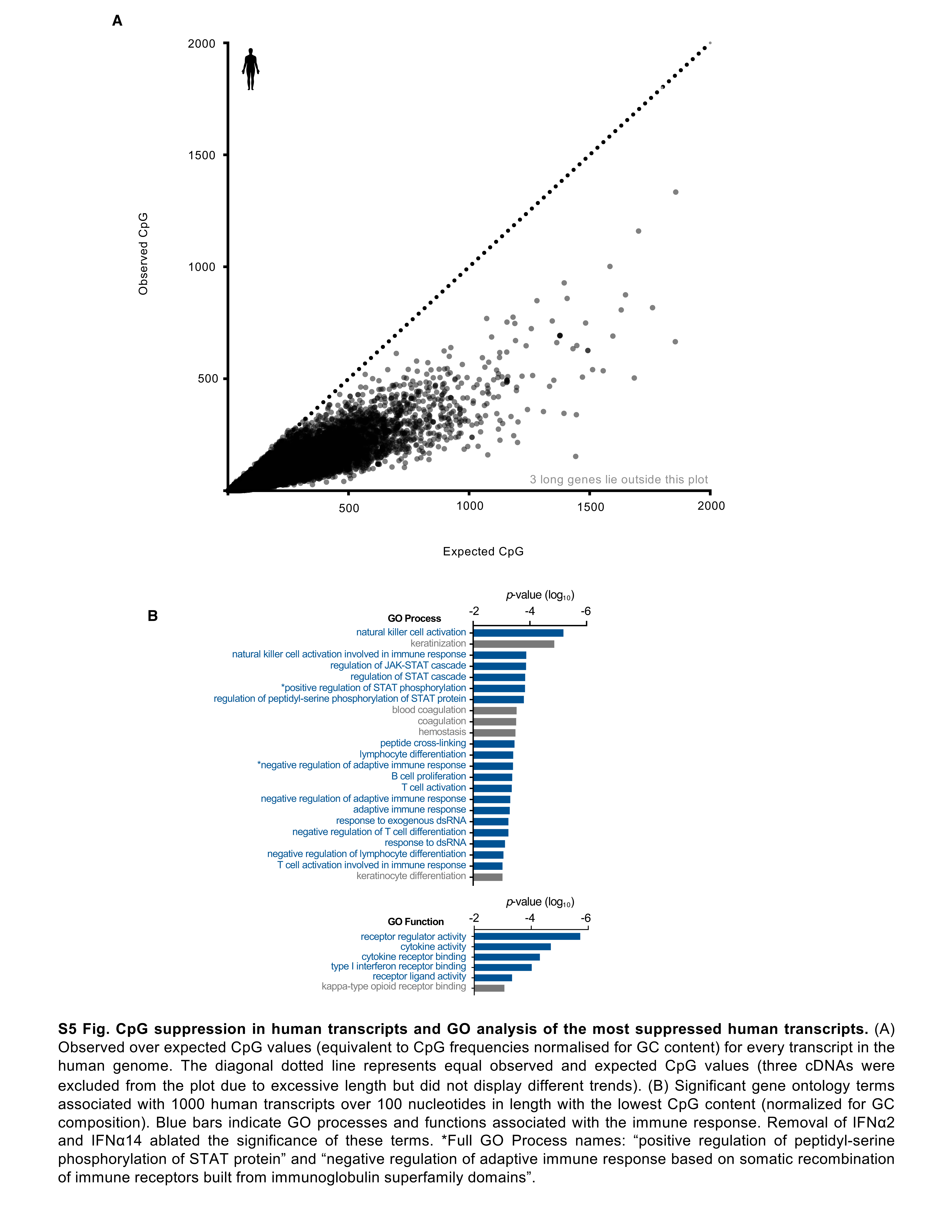

Supplement: S5 Fig — (A) Observed over expected CpG values (equivalent to CpG frequencies normalised for GC content) for every transcript in the human genome. The diagonal dotted line represents equal observed and expected CpG values (3 cDNAs were excluded from the plot due to excessive length but did not display different trends). (B) Significant GO terms associated with 1,000 human transcripts over 100 nucleotides in length with the lowest CpG content (normalised for GC composition). Blue bars indicate GO processes and functions associated with the immune response. Removal of IFNα2 and IFNα14 ablated the significance of these terms. * Full GO process names: “positive regulation of peptidyl-serine phosphorylation of STAT protein” and “negative regulation of adaptive immune response based on somatic recombination of immune receptors built from immunoglobulin superfamily domains”. The underlying data from this figure are openly available (http://dx.doi.org/10.5525/gla.researchdata.1159). GC, guanine–cytosine; GO, gene ontology; IFN, interferon. (TIF) [file pbio.3001352.s005.tif]

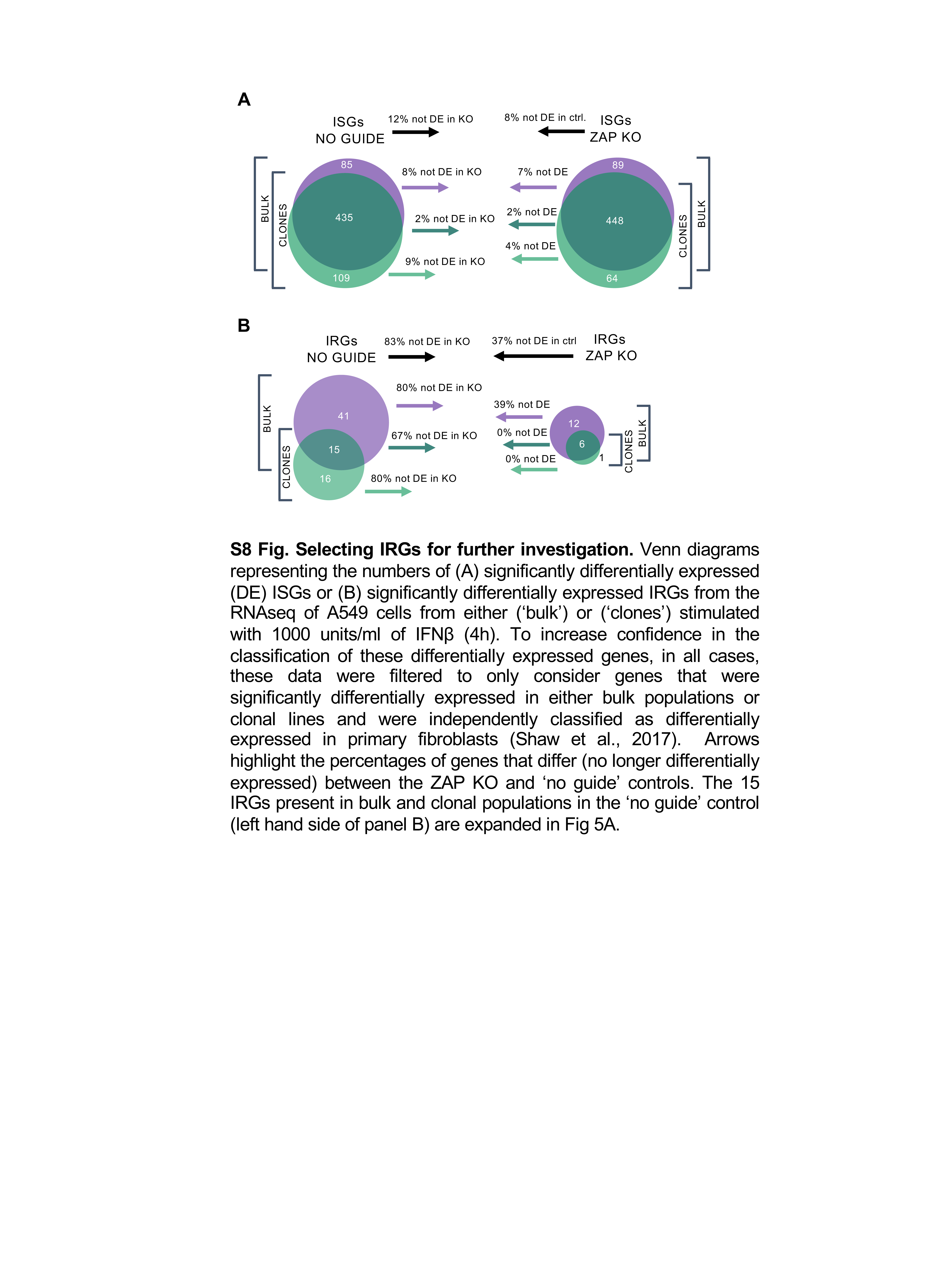

Supplement: S8 Fig — Venn diagrams representing the numbers of (A) significantly DE ISGs or (B) significantly DE IRGs from the RNA-seq of A549 cells from either (“bulk”) or (“clones”) stimulated with 1,000 units/ml of IFNβ (4 hours). To increase confidence in the classification of these DE genes, in all cases, these data were filtered to only consider genes that were significantly DE in either bulk populations or clonal lines and were independently classified as DE in primary fibroblasts [4]. Arrows highlight the percentages of genes that differ (no longer DE) between the ZAP KO and “no guide” controls. The 15 IRGs present in bulk and clonal populations in the “no guide” control (left-hand side of panel B) are expanded in Fig 5A. The underlying data from this figure are openly available (http://dx.doi.org/10.5525/gla.researchdata.1159). DE, differentially expressed; IFN, interferon; IRG, interferon-repressed gene; ISG, interferon-stimulated gene; RNA-seq, RNA sequencing; ZAP, zinc-finger antiviral protein. (TIF) [file pbio.3001352.s008.tif]

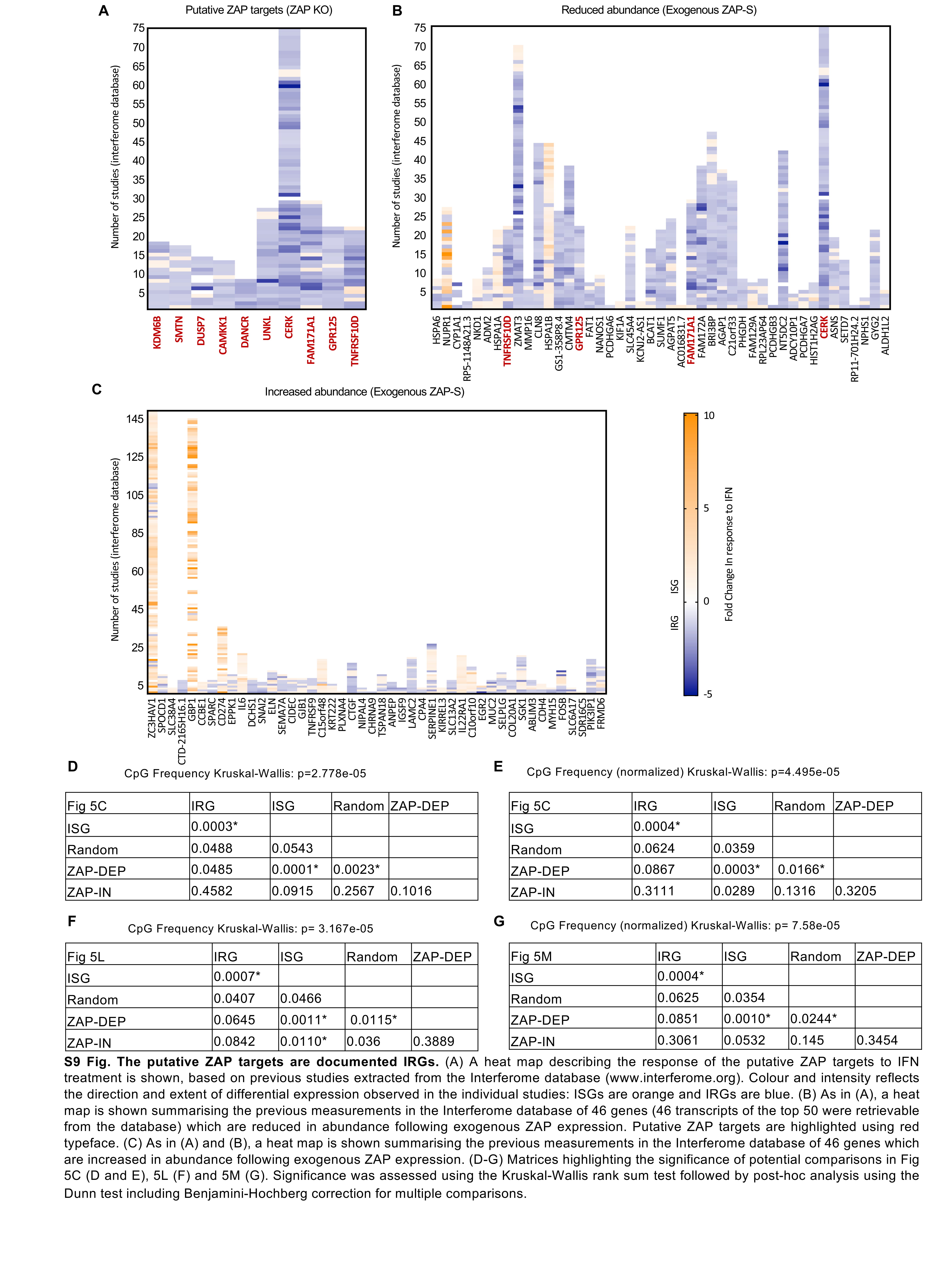

Supplement: S9 Fig — (A) A heat map describing the response of the putative ZAP targets to IFN treatment is shown based on previous studies extracted from the Interferome database (www.interferome.org). Colour and intensity reflects the direction and extent of differential expression observed in the individual studies: ISGs are orange, and IRGs are blue. (B) As in (A), a heat map is shown summarising the previous measurements in the Interferome database of 46 genes (46 transcripts of the top 50 were retrievable from the database), which are reduced in abundance following exogenous ZAP expression. Putative ZAP targets are highlighted using red typeface. (C) As in (A) and (B), a heat map is shown summarising the previous measurements in the Interferome database of 46 genes which are increased in abundance following exogenous ZAP expression. (D–G) Matrices highlighting the significance of potential comparisons in Fig 5C (D and E), 5L (F) and 5M (G). Significance was assessed using the Kruskal–Wallis rank sum test followed by post hoc analysis using the Dunn test including Benjamini–Hochberg correction for multiple comparisons. The underlying data from this figure are openly available (http://dx.doi.org/10.5525/gla.researchdata.1159). IFN, interferon; IRG, interferon-repressed gene; ISG, interferon-stimulated gene; ZAP, zinc-finger antiviral protein. (TIF) [file pbio.3001352.s009.tif]

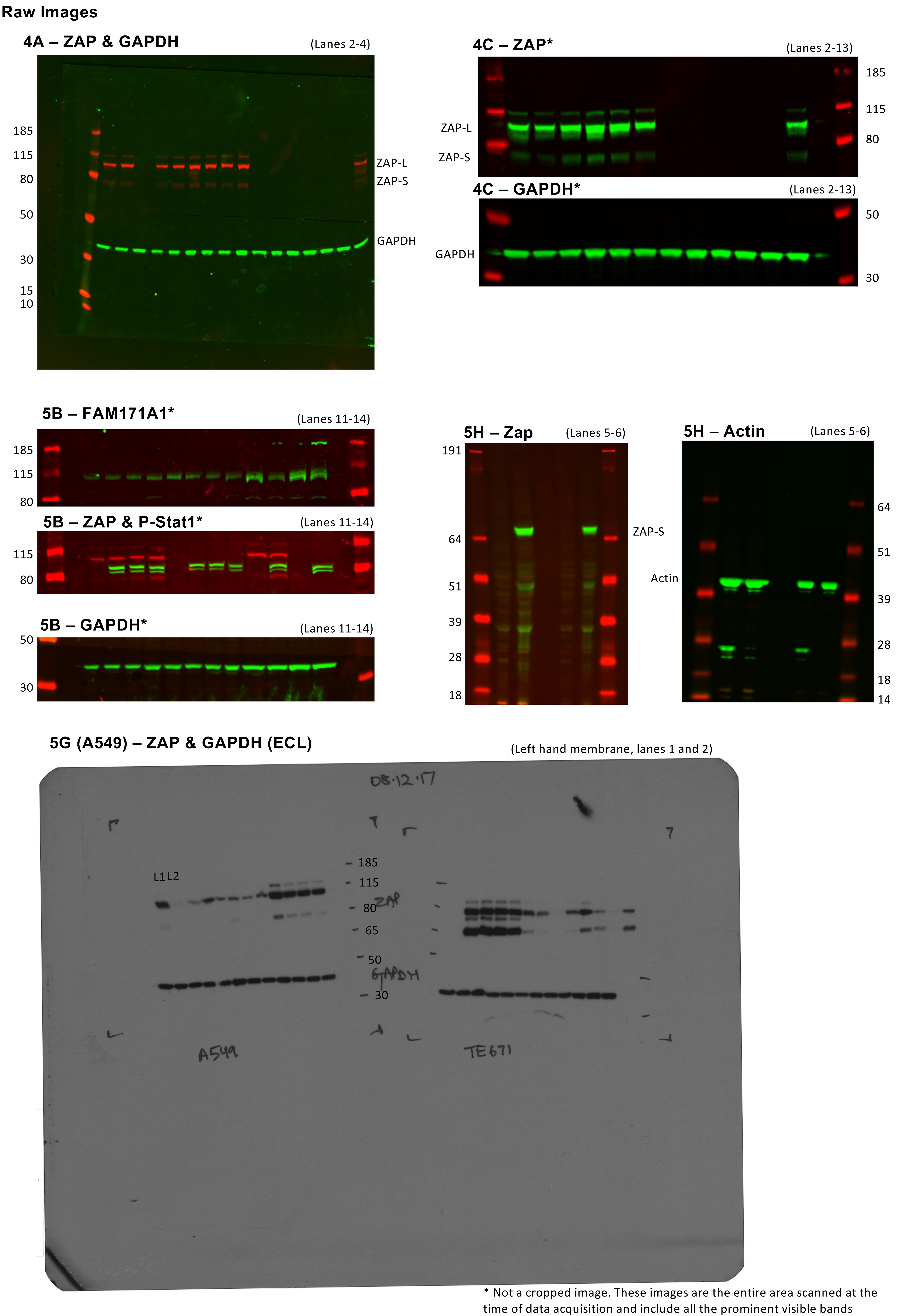

Supplement: S1 Raw Images — The underlying image files are openly available (http://dx.doi.org/10.5525/gla.researchdata.1159). WB, western blot. (TIF) [file pbio.3001352.s012.tif]
